# Modulated anharmonic ADPs are intrinsic to aperiodic crystals: a case study on incommensurate Rb_2_ZnCl_4_

**DOI:** 10.1107/S0108768111013814

**Published:** 2011-05-14

**Authors:** Liang Li, Alexander Wölfel, Andreas Schönleber, Swastik Mondal, Antoine M. M. Schreurs, Loes M. J. Kroon-Batenburg, Sander van Smaalen

**Affiliations:** a Laboratory of Crystallography, University of Bayreuth, 95440 Bayreuth, Germany; b Crystal and Structural Chemistry, Bijvoet Center for Biomolecular Research, Faculty of Science, Utrecht University, Padualaan 8, 3584 CH Utrecht, The Netherlands

**Keywords:** aperiodic crystals, incommensurate modulated structures, MEM density, ADPs

## Abstract

A combination of structure refinements, analysis of the superspace MEM density and interpretation of difference-Fourier maps has been used to characterize the incommensurate modulation of rubidium tetrachlorozincate, Rb_2_ZnCl_4_, at a temperature of *T* = 196 K, close to the lock-in transition at *T*_lock-in_ = 192 K. The modulation is found to consist of a combination of displacement modulation functions, modulated atomic displacement parameters (ADPs) and modulated third-order anharmonic ADPs. Up to fifth-order Fourier coefficients could be refined against diffraction data containing up to fifth-order satellite reflections. The center-of-charge of the atomic basins of the MEM density and the displacive modulation functions of the structure model provide equivalent descriptions of the displacive modulation. Modulations of the ADPs and anharmonic ADPs are visible in the MEM density, but extracting quantitative information about these modulations appears to be difficult. In the structure refinements the modulation parameters of the ADPs form a dependent set, and *ad hoc* restrictions had to be introduced in the refinements. It is suggested that modulated harmonic ADPs and modulated third-order anharmonic ADPs form an intrinsic part, however small, of incommensurately modulated structures in general. Refinements of alternate models with and without parameters for modulated ADPs lead to significant differences between the parameters of the displacement modulation in these two types of models, thus showing the modulation of ADPs to be important for a correct description of the displacive modulation. The resulting functions do not provide evidence for an interpretation of the modulation by a soliton model.

## Abstract 2

The superspace maximum entropy method (MEM) density in combination with structure refinements has been used to uncover the modulation in incommensurate Rb_2_ZnCl_4_ close to the lock-in transition. Modulated atomic displacement parameters (ADPs) and modulated anharmonic ADPs are found to form an intrinsic part of the modulation. Refined values for the displacement modulation function depend on the presence or absence of modulated ADPs in the model.

## 1. Introduction   

The construction of a model-independent electron-density map from phased structure factors is an important application of the maximum entropy method (MEM) in crystallography (Takata, 2008 ▸; van Smaalen & Netzel, 2009 ▸). Analysis of the electron density after such a reconstruction provides the locations of the atoms in the unit cell. As such, the MEM has been used to determine the locations of the metal atoms in endohedral fullerenes (Nishibori *et al.*, 2006 ▸), to obtain information about disorder (multiple positions) in crystal structures (Dinnebier *et al.*, 1999 ▸; Wang *et al.*, 2001 ▸; Samy *et al.*, 2010 ▸) and about anharmonic atomic displacements (Kumazawa *et al.*, 1995 ▸; Bagautdinov *et al.*, 1998 ▸).

The MEM has been generalized towards the determination of the generalized electron density in 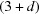-dimensional superspace (*d* = 1, 2, 3,…) of aperiodic crystals (van Smaalen *et al.*, 2003 ▸). Again, the MEM provides information about the locations of the atoms, which then result in a description of the modulation functions of incommensurately modulated crystals or incommensurate composite crystals (Palatinus & van Smaalen, 2004 ▸; van Smaalen & Li, 2009 ▸). Alternatively, the MEM in superspace has been used to determine the occupation domains of the atoms in quasicrystals (Yamamoto *et al.*, 1996 ▸). Here we will use the MEM to obtain information about the modulation functions of incommensurately modulated Rb
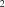
ZnCl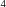.

Many isostructural compounds of the 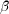-K
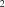
SO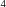 structure type undergo phase transitions on cooling. Several compounds exhibit at least two phase transitions, first forming an incommensurately modulated structure which then becomes commensurate at lower temperatures (lock-in transition; Cummins, 1990 ▸).

**Figure 1:**
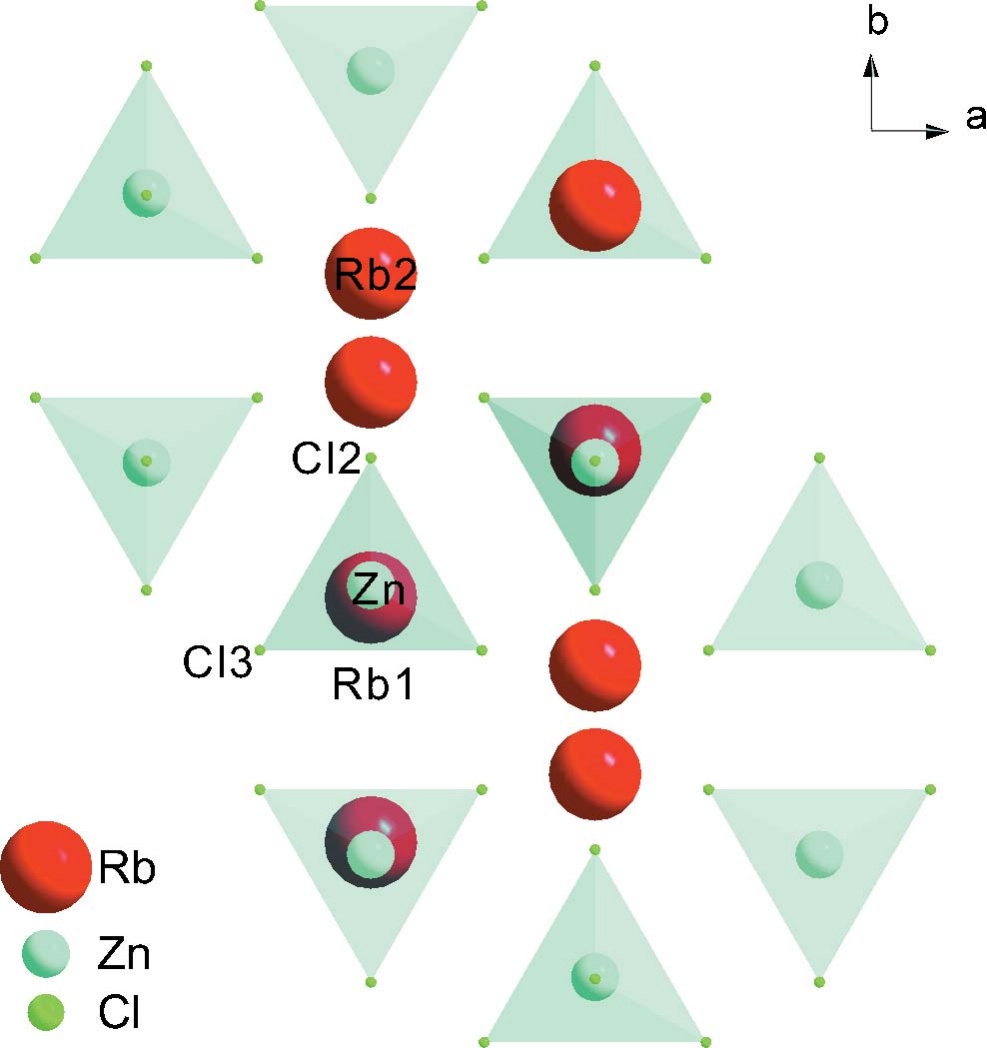
Basic structure of Rb
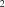
ZnCl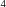. Atoms Rb1, Zn and Cl1 are nearly superimposed in this projection.

Rubidium tetrachlorozincate, Rb
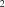
ZnCl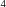, is one of these compounds (Fig. 1 ▸). Rb
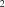
ZnCl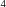 undergoes a phase transition from a periodic to an incommensurately modulated phase at 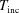 = 303 K. The incommensurate modulation wavevector is 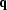 = 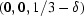 (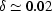). The lock-in transition towards a threefold superstructure (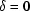) takes place at 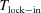 = 192 K (Sawada *et al.*, 1977 ▸).

The modulation of Rb
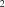
ZnCl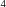 increasingly deviates from a sinusoidal shape on approaching the lock-in transition, as shown by the growth of the intensities of higher-order satellite reflections in the X-ray diffraction of this compound on cooling toward 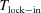 (Aramburu *et al.*, 1997 ▸). The results of structure refinements of a model of displacive modulation functions with up to fifth-order Fourier coefficients have been reported by Aramburu *et al.* (2006 ▸). The latter authors interpreted this structure model as providing evidence for a soliton shape of the incommensurate modulation wave.

Here we present a re-analysis of the incommensurate structure of Rb
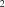
ZnCl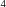 close to the lock-in transition, employing a more extensive data set of Bragg reflections than has been used by Aramburu *et al.* (2006 ▸). The purpose of this work is twofold. The first aim is to investigate the nature of modulations by means of the maximum entropy method (MEM). As we will show, modulations of atomic displacement parameters (ADPs) and modulations of anharmonic ADPs form an intrinsic and important part of the modulation. Secondly we show that the modulation functions do not provide evidence for a soliton character of the modulation in this compound.

## 2. Experimental   

### 2.1. Crystal growth and the diffraction experiment   

Single crystals of Rb
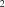
ZnCl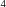 have been grown from aqueous solution (Sawada *et al.*, 1977 ▸). RbCl (2.73 g, Aldrich, 99.99%) and ZnCl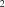 (1.54 g, Aldrich, 99.999%) were dissolved in 4.5 g of ultra pure water (from a Simplicity UV system by Millipore) at *T* = 323 K. The solution was slowly cooled to *T* = 313 K, and crystals were obtained by slow evaporation at this temperature.

A suitable single crystal was glued to a thin glass fibre mounted on a copper pin. X-ray diffraction experiments were performed at beamline F1 of Hasylab, DESY, Hamburg, employing the radiation of a wavelength of 0.5000 Å and a MAR-CCD area detector. The temperature of the sample was maintained at *T* = 196 K, employing a nitrogen-flow cryostat. A large crystal-to-detector distance of 225 mm was chosen, in order to be able to resolve closely spaced reflections.

With the aid of the four-circle kappa diffractometer at beamline F1, diffraction data were collected by 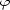 and 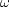 scans with a scan step of 0.3° per image. Several values were chosen for the off-set of the detector and for the orientation of the crystal, thus allowing the measurement of a nearly complete data set up to a high resolution of 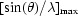 = 0.86 Å^−1^. With the purpose of increasing the effective dynamic range of the experiment, runs with a zero detector off-set were rep­eated with exposure times of 2 and 8 s, and runs at higher scattering angles were repeated with 8 and 64 s exposure. The long exposure times resulted in overexposed strong (main) reflections, while they allowed weak reflections (mostly higher-order satellite reflections) to be measured.

Integrated intensities of Bragg reflections were extracted from the measured images by the software *EVAL*15 (Schreurs *et al.*, 2010 ▸). Absorption correction was performed with *SADABS* (Sheldrick, 2008 ▸). A fraction of the area of the CCD detector was not properly cooled during parts of the experiment. This is a technical problem that occurred for experiments of long durations (Paulmann, 2009 ▸). As a result several pixels of the detector always gave a large intensity, which could negatively affect data quality. Therefore, the coordinates of these pixels have been determined by inspection of the images, and they were excluded from the integration. Experimental data and crystallographic information are summarized in Table 1 ▸.^1^ The observed volume of the unit cell is significantly smaller than reported by Aramburu *et al.* (2006 ▸), who gave 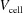 = 844.04 Å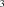 with *a* = 7.241 (3), *b* = 12.648 (5) and *c* = 9.216 (3) Å. Since lattice parameters from point-detector measurements are much more accurate than from area detectors, we have employed the lattice parameters from Aramburu *et al.* (2006 ▸) in the present refinements.

The resulting data set of intensities of Bragg reflections — including satellite reflections up to fifth order — was used for structure determination, structure refinements and maximum entropy calculations.

Aramburu *et al.* (2006 ▸) have kindly supplied the diffraction data from their publication. These data will be denoted as the Aramburu data. Various models have also been tested by calculation of the values of 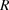 indices on these data.

A peculiar property of the Aramburu data is that a selection of the reflections were measured, which included all main reflections and only the strongest satellite reflections as expected on the basis of a soliton model. Satellite reflections up to order five, except fourth order, have been measured in this way by Aramburu *et al.* (2006 ▸). The result is a data set that consists of many fewer reflections than available in the present data. On the other hand, CCD detectors have a limited dynamic range so that the lower bound on measurable intensities is relatively high, resulting in the number of high-order ‘observed’ satellite reflections being comparable in the two data sets (Table 1 ▸).

### 2.2. Structure refinements   

Structure models of different complexity have been refined against the diffraction data. They involve the basic structure coordinates 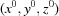 and the harmonic atomic displacement parameters (ADPs) 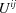 for each of the six crystallographically independent atoms (Fig. 1 ▸). Depending on the complexity of the model, they may include Fourier coefficients for displacement modulation (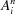 and 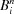 for the sine and cosine Fourier coefficients of the order 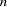 along the direction 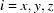); anharmonic ADPs of third (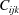) and fourth (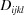) order; Fourier coefficients for the modulation of the ADPs (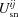, 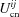 for the sine and cosine Fourier coefficients of order 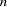) as well as 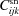 and 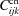 (Table 2 ▸; van Smaalen, 2007 ▸).

Structure refinements were performed with the computer program *JANA*2006 (Petricek *et al.*, 2006 ▸). The model published by Aramburu *et al.* (2006 ▸) involves displacement modulation parameters of orders 1, 2, 3 and 5. Refinement of these parameters against the Aramburu data reproduced the published model within one standard uncertainty (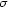) of all parameters.

Model A was created to resemble the published structure model as much as possible. It includes all Fourier coefficients up to fifth order for the displacement modulation, because the availability of fourth-order satellite reflections in the present data allows the refinement of the fourth-order Fourier coefficients of the displacement modulations. Refinements were initiated with the values of the published structure model as starting parameters. Values of the refined parameters are similar to those of the published structure model, with only 12 out of 140 parameters having differences larger than 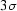 and with a maximum difference of 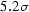 for 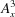 of atom Rb1 (*cf.* Table 3 ▸ with Table VI in Aramburu *et al.*, 2006 ▸).

Model B is an extension of model A, where the first- and second-order Fourier coefficients of the modulation of the harmonic ADPs have been incorporated. Refinements with model A as starting values for the parameters gave a smooth convergence and led to a considerable improvement of the fit to all orders of reflections (Table 4 ▸).

Model B was used to create the phased observed diffraction data from the measured intensities for the MEM calculations (see §2.3). Analysis of the MEM-derived electron-density map suggested that the next important feature was the modulation of the third-order anharmonic ADPs, while their average structure values remained zero. Model C includes, in addition to the parameters of model B, the Fourier coefficients up to 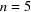 for the modulation of the third-order anharmonic ADPs, 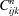. This refinement suffered from large correlations between parameters. Therefore, a reduced model, model C_r_, was defined, in which those Fourier coefficients 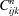 were set to zero that had values less than 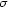 in the refinement of model C. This reduced the number of coefficients 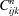 from 244 to 132 (Table 2 ▸), while models C and C_r_ fitted the data almost equally well (Table 4 ▸).

**Figure 2:**
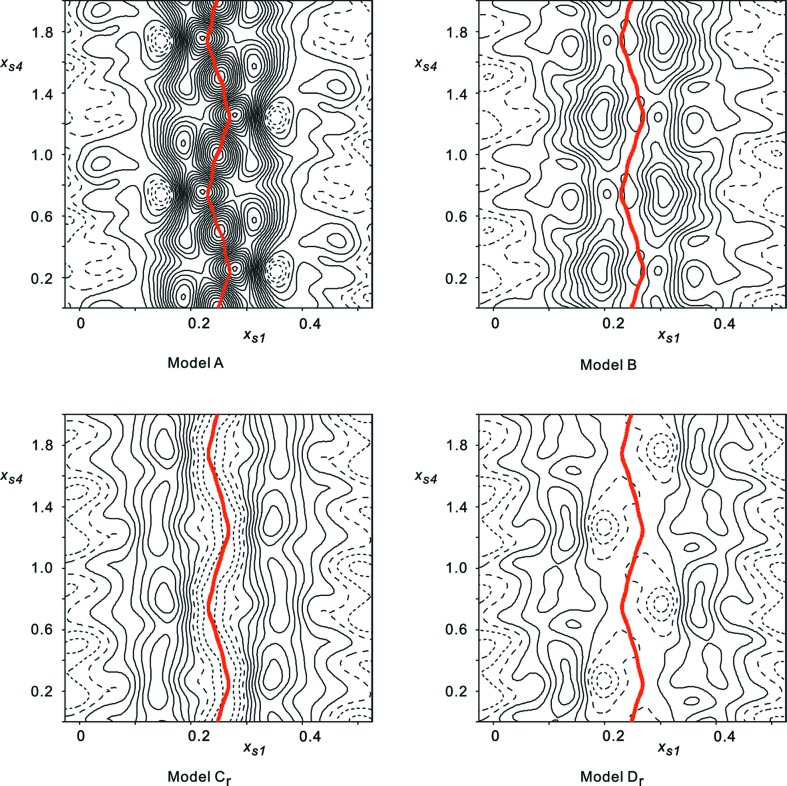
(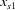, 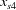)-sections of difference-Fourier maps centered on the Rb2 atom (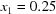, 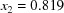 and 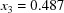) from different models, showing two periods along 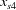. Solid lines represent positive values, dashed lines are negative values, and long dashed lines represent the zero contour. The contour interval is 0.2 e Å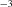. Maximum and minimum values over the map are 3.63/−0.57 e Å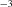 for model A, 2.12/−0.41 e Å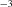 for model B, 1.35/−0.52 e Å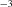 for model C_r_, 0.93/−0.51 e Å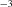 for model D_r_. The thick (red) lines denote the modulated position of the Rb2 atom.

Difference-Fourier maps based on the observed structure factors and those calculated for a model indicate the improvement of the fit to the data for increasing complexity of the model (Fig. 2 ▸ and Table 4 ▸). The difference-Fourier map of model B compared with that of model C_r_ confirms the importance of modulated third-order anharmonic ADPs, as it has been derived based on MEM density maps. The difference-Fourier map of model C_r_ displays structure around the Rb2 atom which, to a first approximation, is independent of the phase of the modulation. It has the signature of unmodulated fourth-order anharmonic ADPs, as they are missing in model C_r_. The inclusion of fourth-order anharmonic ADPs for all atoms led to highly nonphysical values of these parameters, that is, large negative values of the joint probability distribution function for the resulting model. Model D_r_ was then constructed to include fourth-order anharmonic ADPs for atoms Rb1, Rb2 and Cl3 only. The improvement, compared with model C_r_, of the fit to the data, in particular to the main reflections, is apparent (Table 4 ▸). Refinements of the extinction coefficient led to a negative value for this parameter, so it was fixed to zero.

The remaining discrepancies between calculated and observed structure factors can be attributed in part to the incompleteness of the model. As indicated above, the introduction of more parameters leads to nonphysical values and high correlations between them, while these additional parameters would have been required for a full characterization of the modulation. A second reason for the rather high final 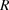 values of the higher-order satellite reflections lies in the less than optimal accuracy of the present data due to limited counting statistics. This interpretation becomes apparent when the 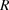 values are considered for model D_r_ on the stronger reflections of the present data [reflections with 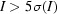; column 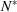 in Table 4 ▸]. In particular, the partial 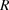 values of the higher-order satellite reflections are considerably lower than on the full data set (compare columns D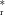 and D_r_ in Table 4 ▸).

The fit of the models A, B, C, C_r_ and D_r_ to the Aramburu data has been tested by refinement of the basic structure parameters of each model against these data, while the modulation parameters and anharmonic ADPs were kept fixed to the values determined from the present data. The fit to the main reflections and first-order satellite reflections is reasonable, but it becomes worse on the introduction of modulation parameters for the (an)harmonic ADPs (models B–D_r_; Table 5 ▸). On the other hand, the latter models lead to an improvement of the fit to the third- and fifth-order satellites of the Aramburu data, but with 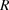 values that are considerably higher than those on the present data. These discrepancies can be attributed to different qualities of the sample and especially different temperatures, which will affect the shapes of the modulation functions and the contributions of modulated and anharmonic ADPs to it.

Therefore, independent refinements were performed against the Aramburu data, now varying all parameters, and resulting in models A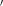, B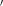, C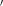, C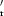 and D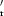, which differ from the corresponding models A, B, C, C_r_ and D_r_ in the values of the parameters. The fit to the Aramburu data is dramatically improved in this way (see supplementary material), resulting in 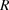 values comparable to 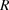 values on the present data. Exceptions are the main reflections, which are much better fitted for the present data, indicating the higher accuracy of these data compared with the Aramburu data.

Despite convergence of the refinements against the Aramburu data and the resulting low 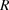 values, the primed models suffer from high correlations between parameters and large standard uncertainties. For example, none of the modulation parameters for ADPs in model B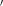 exceed 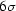, which prevents a meaningful analysis of the modulation on the basis of model B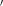, as has already been noted by Aramburu *et al.* (2006 ▸).

The standard uncertainties of modulation parameters and anharmonic ADPs are a multiple of the standard uncertainties of these parameters in the corresponding unprimed models (refinements against the present data). Therefore, we refrain from a further consideration of the primed models.

### 2.3. MEM calculations   

Phased observed structure factors corrected for anomalous scattering and scaled to the scattering power of one unit cell were obtained from the observed data and model B according to published procedures (Bagautdinov *et al.*, 1998 ▸). These data were used for the calculation of a maximum-entropy-optimized generalized electron density in 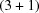-dimensional superspace [MEM density or 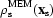] with the computer program *BAYMEM* (van Smaalen *et al.*, 2003 ▸). A uniform prior, the Cambridge algorithm and the weights of type F2 have been used (Li *et al.*, 2010 ▸). The MEM calculation converged in 69 iterations (see Table 1 ▸ for more information on the MEM calculation).

The 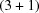-dimensional electron-density map has been analyzed with the computer program *EDMA* (van Smaalen *et al.*, 2003 ▸). Physical space sections of 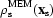 have been obtained for 100 equally spaced 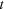 values within one period along the fourth axis, *i.e.* for 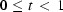. Atoms in the crystal correspond to local maxima in the physical space sections of the generalized electron density.

**Figure 3:**
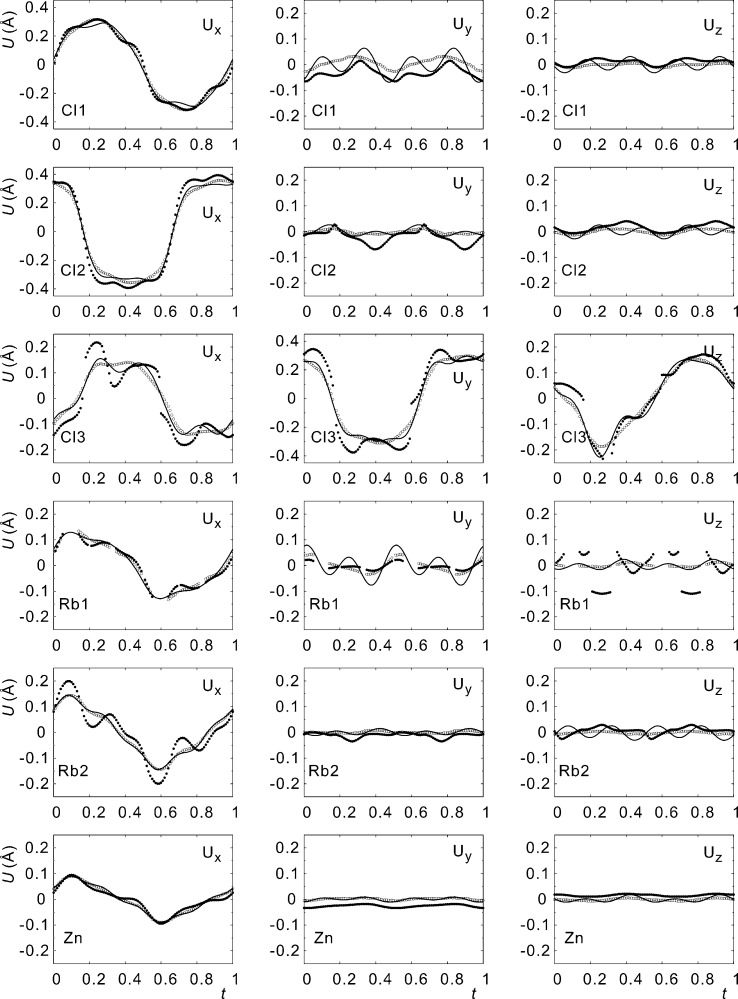
Modulation functions of the crystallographically independent atoms of Rb
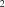
ZnCl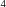. Displacements along 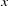, 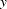 and 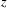 are given in Å. Open circles represent the center of the charge and filled circles are the maxima of the MEM electron density. Lines represent the modulation functions of model A.

The position of each local maximum as a function of 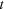 then provides an estimate for the modulated position of an atom. Alternatively, the center-of-charge has been determined for the atomic basins surrounding each local maximum. The dependence on 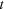 of the positions of the center-of-charge provides an alternative measure for the atomic positions. Modulation functions have been extracted from 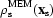 by taking the difference between the modulated atomic position and the basic structure position as obtained from model B (Fig. 3 ▸).

**Figure 4:**
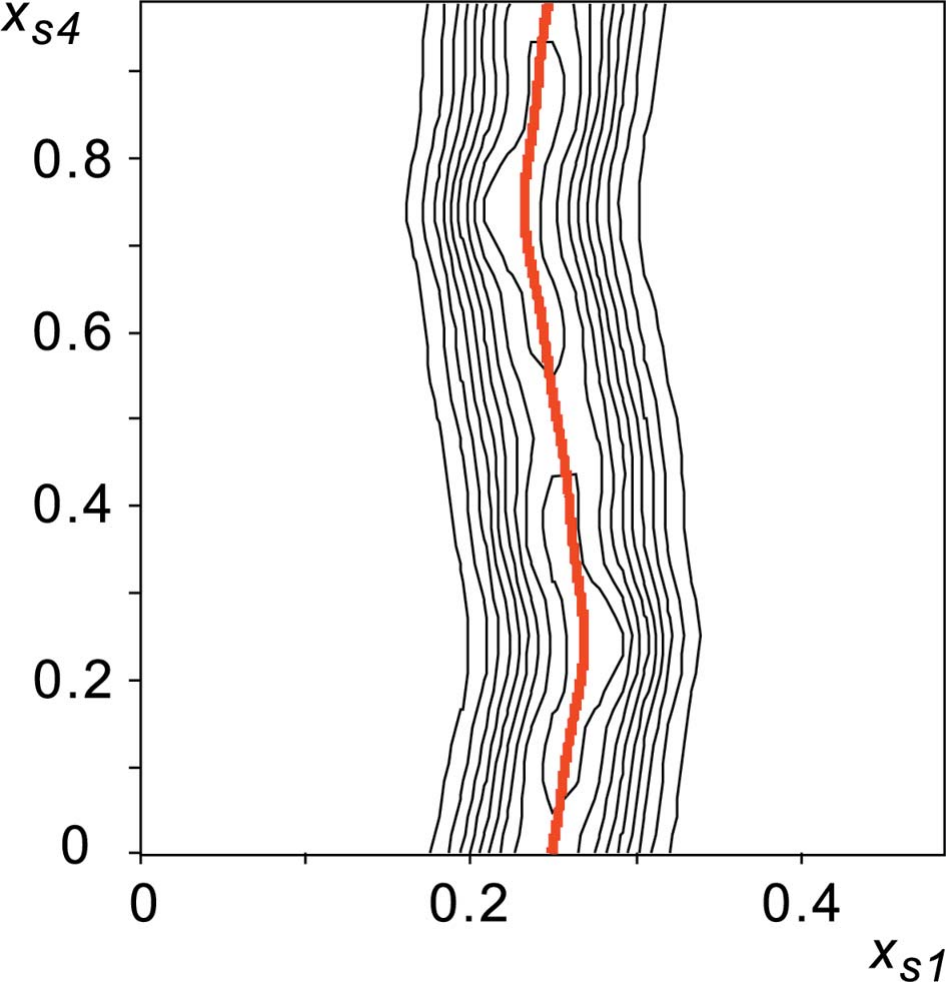
(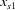, 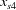)-section of the generalized electron density 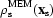 at the position of Rb2 (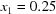, 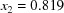 and 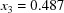). The contour interval is 10% of the maximum electron density of 175.1 e Å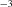. The thick (red) line is the modulated position of the Rb2 atom in model D_r_.

Two-dimensional sections of 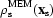 have been visualized by the plotting option of the computer program *JANA*2006 (Petricek *et al.*, 2006 ▸). The (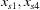) section centered on the Rb2 atom clearly shows the modulated position of this atom (Fig. 4 ▸).

## 3. Discussion   

### 3.1. Nature of the modulation   

The incommensurate modulation of Rb
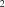
ZnCl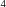 at a temperature of *T* = 196 K, close to the lock-in transition at 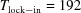 K, has been determined to be comprised of atomic displacement modulation functions that contain contributions of Fourier coefficients up to fifth order. This finding is in agreement with previous studies on this compound (Aramburu *et al.*, 2006 ▸).

Modulations of the ADPs are found to be an intrinsic part of the incommensurate crystal structure. That is, the harmonic ADPs are modulated with up to second-order Fourier coefficients and the third-order anharmonic ADPs are modulated with Fourier coefficients up to fifth order, while the basic structure components of the third-order anharmonic ADPs are zero.

The finding of modulated ADPs is in agreement with studies on several other compounds, such as incommensurately modulated Na
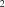
CO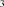 (Dusek *et al.*, 2003 ▸), and the composite crystals [LaS]
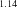
[NbS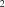] and [SrO]
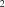
[CrO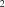]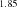 (Jobst & van Smaalen, 2002 ▸; Castillo-Martínez *et al.*, 2008 ▸). Modulated ADPs have also been found necessary in cases where only first-order satellite reflections were available in the diffraction data, like La
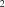
C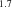, Na
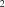
Si
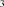
O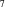 and Pb
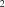
NiVO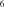 (Dusek *et al.*, 2000 ▸; Krüger *et al.*, 2006 ▸; Roussel *et al.*, 2009 ▸).

The necessity of modulation functions for third-order anharmonic ADPs has been revealed in our previous studies with the MEM on (NH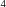)
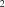
BeF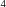 (Palatinus *et al.*, 2004 ▸) and Cr
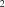
P
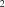
O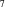 (Li *et al.*, 2010 ▸). Many incommensurate crystal structures have been published, where 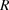 values are higher than they should have been for the perfect structure model. It can thus be speculated that the fit to diffraction data might be improved for many compounds by the inclusion of modulated ADPs and modulated third-order anharmonic ADPs. On the other hand, correlations between modulation parameters, as shown here for Rb
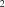
ZnCl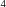, might prevent their determination by structure refinements. This problem especially exists for high-order Fourier coefficients of modulation functions. Meaningful values are almost always limited to coefficients of orders equal to and less than the maximum order of observed satellite reflections.

It is suggested here that modulations of ADPs are an intrinsic part of modulations in incommensurate crystals in general. Their presence can be rationalized by the fact that any displacive modulation defines a modulation of the environments of the atoms. Different environments require different ADPs, which can be achieved by a modulation of the ADPs. It is important to include at least up to second-order Fourier coefficients (Perez-Mato *et al.*, 1991 ▸).

The displacement modulation of an atom creates a tightening of its environment in the direction in which this atom is displaced. Since the modulation always involves displacements out of the average position into both the positive and negative directions, this general feature of modulations explains the presence of modulated third-order anharmonic ADPs, while their average values are zero. The correlation between displacement modulation and modulated 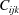 is apparent from the values found for model D_r_ of Rb
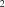
ZnCl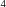. Both the displacement modulation and the modulated 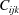 have their most important nonzero components along the 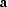 axis for all five independent atoms on the mirror plane (Table 6 ▸ and supplementary material). The Cl3 atom, not on the mirror plane, also has contributions to its modulation for the other directions, which again affects both the displacement modulation and the modulated third-order anharmonic ADPs.

**Figure 5:**
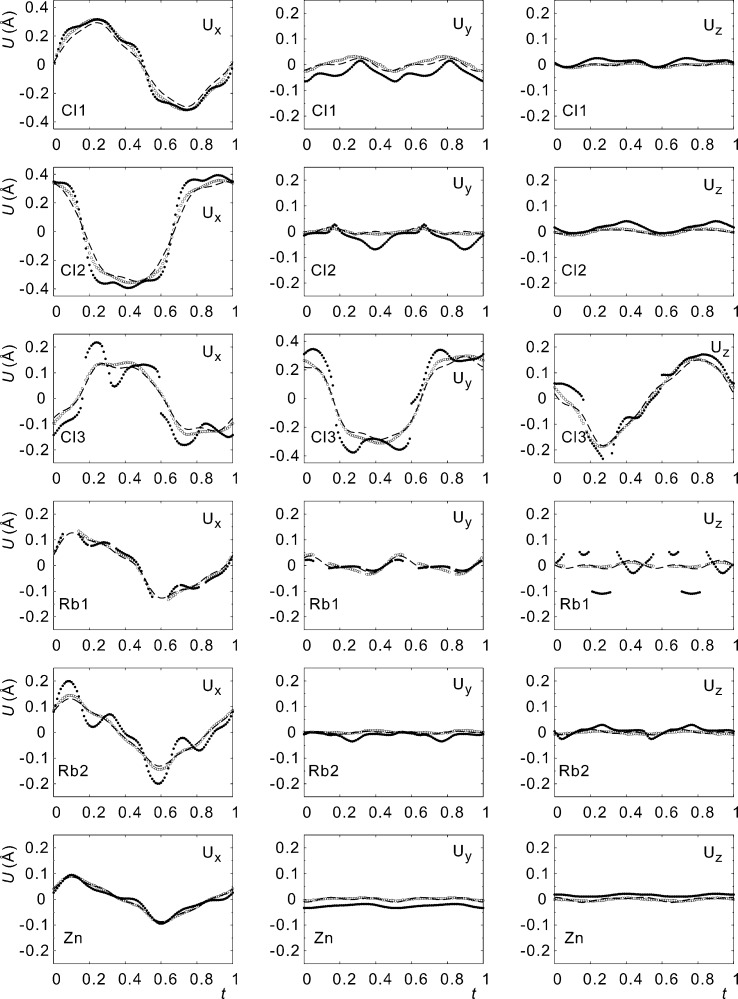
Modulation functions of the crystallographically independent atoms of Rb
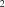
ZnCl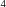. Displacements along 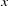, 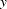 and 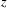 are given in Å. Open circles are the center of the charge and filled circles are the maxima of the MEM electron density. Dashed lines represent the modulation functions of model D_r_.

**Figure 6:**
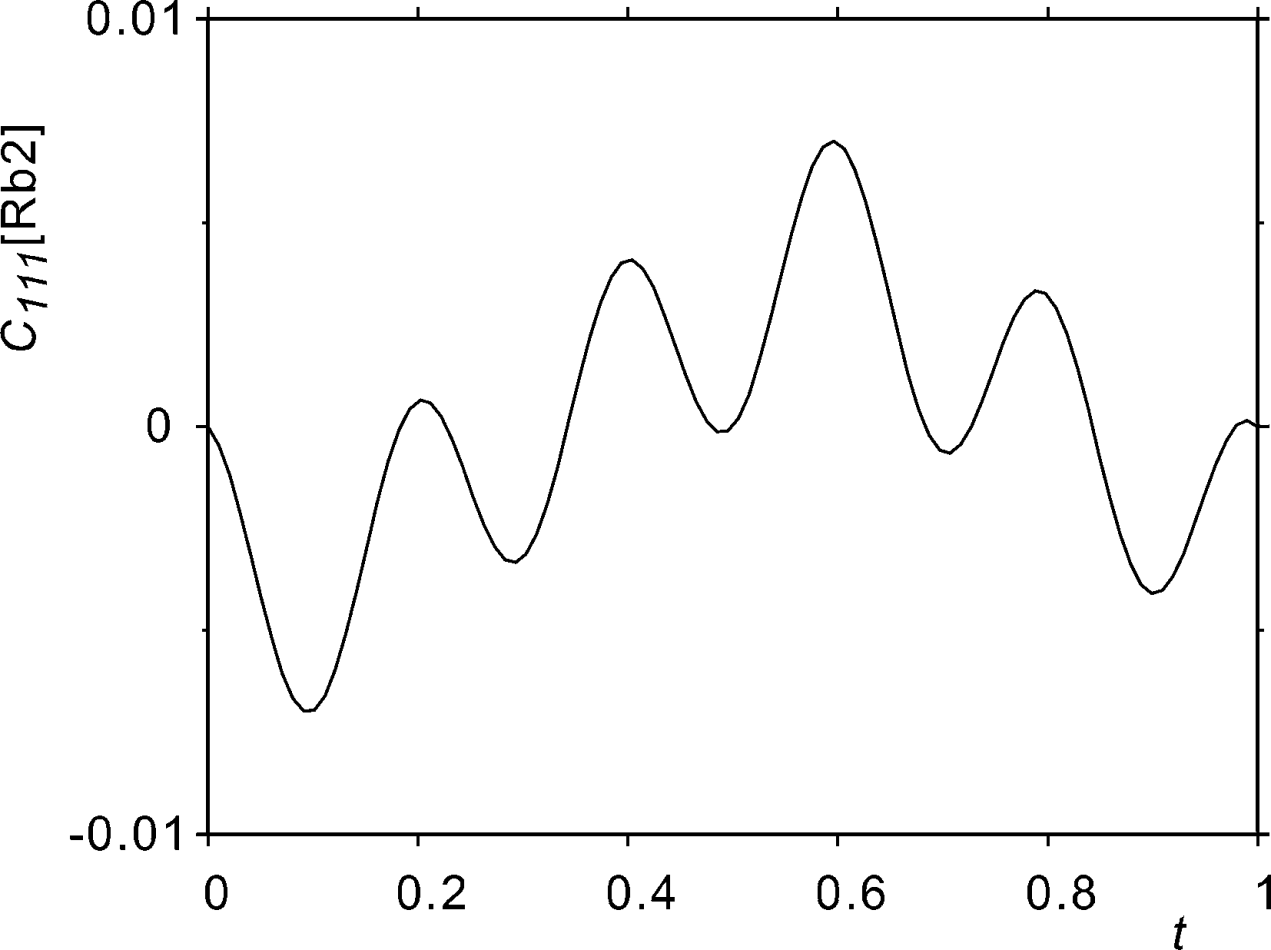
Value in model D_r_ of the component 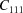 of third-order anharmonic ADPs of the Rb2 atom as a function of 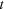. Minimum and maximum values are located at 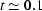 and 0.6.

**Figure 7:**
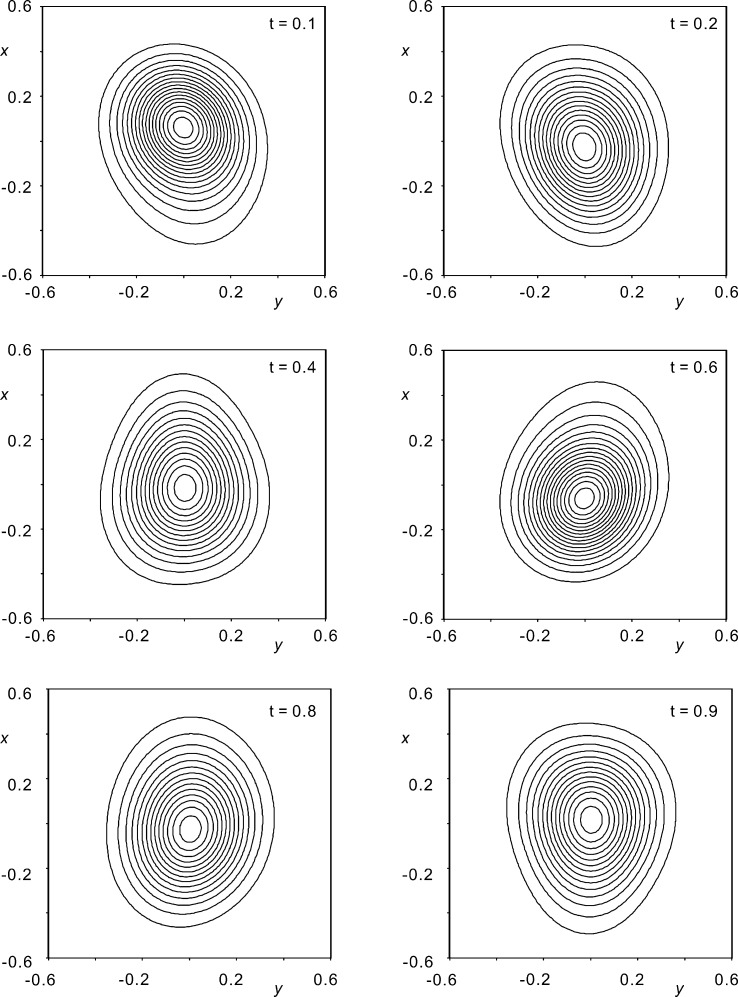
The joint probability distribution function at the site of Rb2 for selected 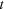 values for model D_r_, but obtained with 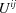 and 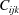 only. The contour interval is 1 e Å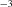 with a maximum density of 17.7 e Å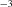.

Although not perfectly matched, positive displacements along 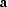 (Fig. 5 ▸) of the Rb2 atom match negative values of 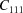 (Fig. 6 ▸), which implies a decreased probability (Figs. 7 ▸ and 8 ▸) for the presence of an atom in the direction of a tighter environment.

The trace of the center-of-charge for each atom in the MEM density indicates smooth modulations, whereas the trace of the local maximum of the density varies around the position defined by the center-of-charge for each atom (Fig. 3 ▸). We take this variation as evidence for the presence of modulated third-order anharmonic ADPs. Similarly, several of the components of the modulation functions in model A exhibit variations (ripples) according to higher-order Fourier coefficients, while the same functions appear smooth in model D_r_ (Fig. 5 ▸). (The largest effect is visible for the components 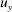
[Cl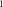], 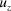
[Cl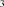] and 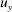
[Rb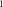].) These smoother functions seem more plausible and they match the trace of the center-of-charge of the MEM density very well. Both refinements (model D_r_ *versus* model A) and the MEM thus provide evidence for modulated third-order anharmonic ADPs. Lastly, the reduction of 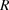 values on the increasing complexity of the structure model provides strong evidence for modulated harmonic ADPs and modulated third-order anharmonic ADPs (Table 4 ▸).

As mentioned above, structure refinements without (model A) and with modulation functions for ADPs (models B–D_r_) result in significantly different functions for the displacive modulation. The inclusion of modulation functions for ADPs thus appears to be necessary to achieve an accurate description of the displacive modulation, with concomitant implications for the interpretation of the modulation (§3.2). Alternatively, the center-of-charge of each atom in the MEM density also provides a good description of the displacement modulation functions.

Comparison of the two approaches, MEM analysis and structure refinements, shows the different limitations of the two methods. The MEM density gives evidence for the modulations of the ADPs as well as the presence of anharmonic ADPs. However, the finite size of the pixels in the MEM density (here 0.1 Å) limits the accuracy of the atomic positions to ∼ 0.01 Å (van Smaalen *et al.*, 2003 ▸), while atoms on special positions may sometimes lead to more accurate values of the positions. An error of up to 0.01 Å is not small, if modulations are considered with amplitudes significantly below 0.1 Å. On the other hand, structure refinements readily lead to large dependencies between parameters, such that Fourier components of orders 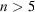 cannot be determined. Furthermore, a full *ab initio* determination appeared impossible for the third- and fourth-order anharmonic ADPs, and we had to resort to a method of selecting relevant parameters (compare models C, C_r_ and D_r_ and the discussion in §2.2).

### 3.2. Relation to the soliton model   

Aramburu *et al.* (2006 ▸) have shown that a soliton model for the modulation leads to displacement modulation functions with Fourier components of first and fifth (and higher) order. They introduced a measure, 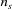, for the soliton density, which can be interpreted as the ratio between the width and the separation of the discommensurations (Babkevich & Cowley, 1999 ▸), and which describes the shape of the modulation functions with 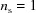 for a sinusoidal shape and 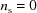 for non-overlapping discommensurations. Within this approach, the phase of the fifth-order Fourier coefficient depends on the phase of the first-order Fourier coefficient by a simple relation and it is independent from the soliton density. The ratio between amplitudes of fifth- and first-order Fourier coefficients should be the same for all atoms, while its value is a measure for the soliton density. Aramburu *et al.* (2006 ▸) have found these relations to be approximately valid for their structure model for Rb
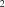
ZnCl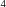, and they proposed that the modulation of Rb
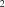
ZnCl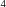 can be described by a soliton wave, with a soliton density of 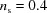 at the temperature of their experiment.

**Figure 9:**
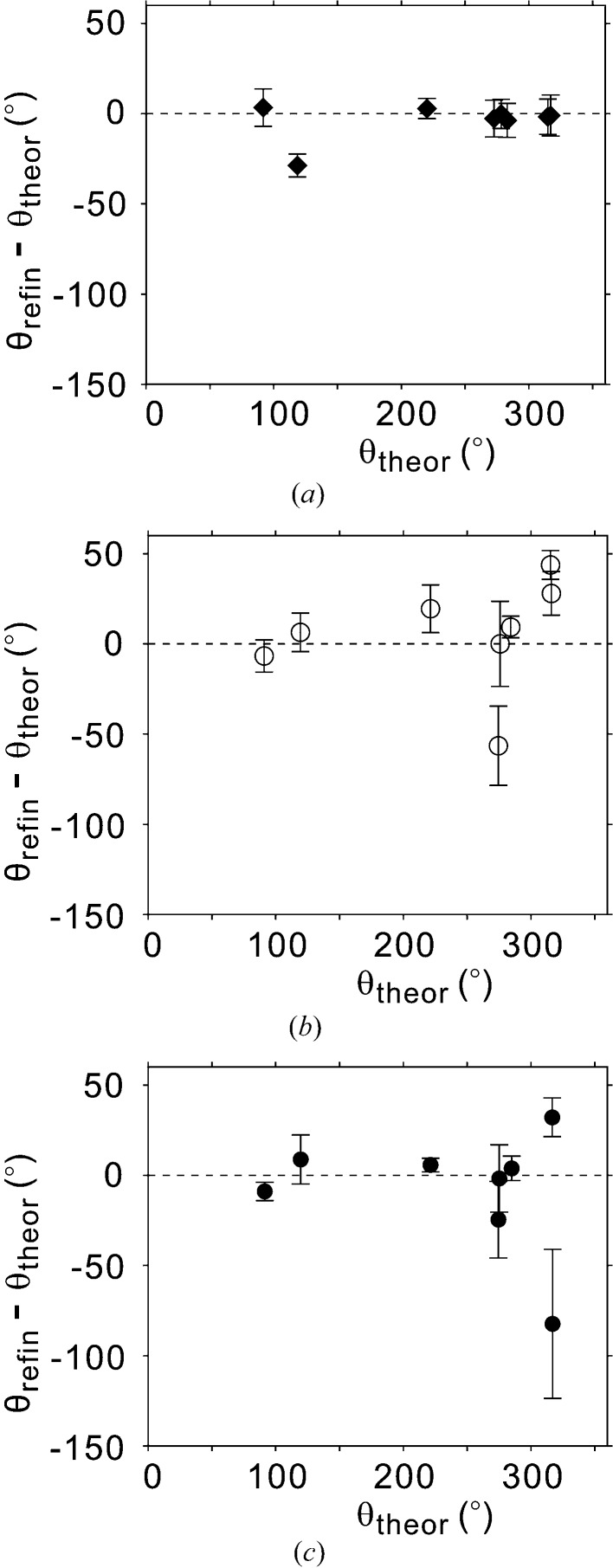
Difference between experimental and calculated values of the phases of the fifth-order Fourier coefficients of the displacive modulation functions for model A (open circles), model D_r_ (filled circles) and the Aramburu *et al.* (2006 ▸) model (diamonds). 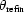 is obtained from the refined fifth-order coefficients. 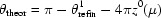 is calculated from the phases (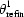) of the refined first-order Fourier coefficients of the same models according to the relation obtained for the soliton model by Aramburu *et al.* (2006 ▸). Error bars indicate one standard uncertainty.

Here we have shown that significant differences exist for the displacement modulation functions in cases of a pure displacive modulation model (model A) and a model including modulated (an)harmonic ADPs (model D_r_). Since model D_r_ is the more accurate model and the Aramburu model resembles model A, this finding questions the interpretation by Aramburu *et al.* (2006 ▸) concerning the soliton shape of displacive modulations in their model. The data from Aramburu *et al.* (2006 ▸) are re-plotted in Figs. 9 ▸ and 10 ▸. The relation between the phases of the first- and fifth-order Fourier coefficients matches that of a soliton wave much better than the observed standard uncertainties would suggest. This excellent agreement might be an artefact resulting from the fact that the only reflections included in the data set were those which possessed high intensities in the soliton model. On the other hand, one out of eight data points has a phase relation that is significantly different from the soliton model, again suggesting that the Aramburu data do not necessarily provide evidence of the soliton model.

**Figure 10:**
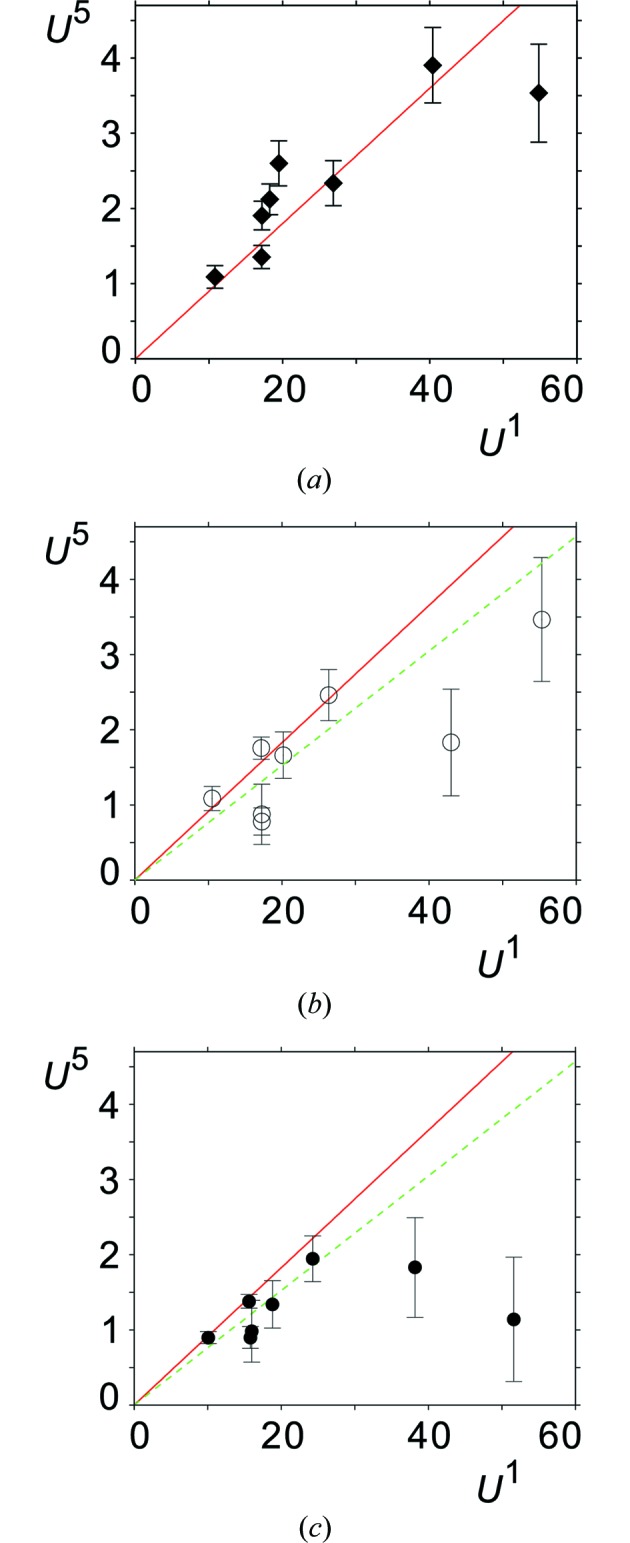
Relation between the amplitudes (multiplied by 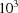
 Å) of the fifth- (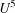) and first-order (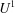) Fourier coefficients of the modulation functions of model A (open circles), model D (filled circles) and the model from Aramburu *et al.* (2006 ▸) (diamonds). The soliton model would require all points to lie on a straight line with a slope that defines the soliton density. The solid line is calculated for a soliton density of 0.4 proposed in Aramburu *et al.* (2006 ▸); the dashed line has been obtained by a least-squares fit to the values of model D_r_ and it represents a soliton density of 0.45. Error bars indicate one standard uncertainty.

Both models A and D_r_ appear to be at variance with the soliton model, as follows most prominently from the ratio of amplitudes of fifth- and first-order Fourier coefficients (Fig. 10 ▸). The standard uncertainties of the phases of the fifth Fourier coefficients are much larger for some functions in models A and D_r_ than in the Aramburu model. While standard uncertainties of refined parameters are of comparable magnitude in the different models, this discrepancy can be ascribed to the much smaller amplitudes of some fifth-order coefficients in model D_r_ than in the Aramburu model, thus leading to a poorer estimate of the phases of these functions. Nevertheless, large discrepancies are found between the calculated and experimental phases of the fifth-order Fourier coefficients, again amounting to several standard uncertainties. This indicates that the present data fail to provide evidence for a soliton shape of the modulation functions.

Another feature of the modulation arguing against the soliton model is the presence of Fourier coefficients of orders two, three and four, with magnitudes comparable to the magnitudes of the fifth-order coefficients (Table 6 ▸). The interpretation favored by Aramburu *et al.* (2006 ▸) is that the third-order Fourier coefficient would represent a secondary mode, while they have not determined the fourth-order Fourier coefficients and the second-order Fourier coefficients are much smaller in the model by Aramburu *et al.* (2006 ▸) than presently obtained (Table 6 ▸). The interpretation of Aramburu *et al.* (2006 ▸) would thus imply that secondary modes are more important than the distortion (magnitude of fifth harmonics) toward the supposed soliton-shaped wave, a situation that is not necessarily likely.

An alternative interpretation of the observed structure model is that of a squaring of the modulation wave, then involving all harmonics of the modulation functions (Leist *et al.*, 2008 ▸). This interpretation is not at variance with the interpretation of the incommensurate structure by discommensurations. It only questions the structure of the discommensurations as a structure given by the solution of the sine-Gordon equation. Since the sine-Gordon equation is only a simple model for discommensurations, a more advanced theory might be able to describe the observed shapes of the modulation functions.

Finally, we have presently established that modulated harmonic ADPs and modulated third-order anharmonic ADPs are an important part of the modulation. However, these features have not been incorporated into the soliton model considered by Aramburu *et al.* (2006 ▸). Establishing the relation between modulated ADPs and a possible soliton property of the modulation wave will require further theoretical analysis that is beyond the scope of the present work.

### 3.3. Origin of the modulation   

The origin of the modulations in Rb
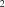
ZnCl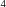 and in *A* 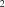 *BX* 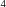-type compounds in general lies in the incompatibility between the observed orthorhombic packing of ZnCl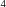 and Rb ions and the nearly tetrahedral symmetry of the ZnCl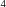 complex ions. This incompatibility results in one short distance between the 11-coordinated A atom (Rb1 in the present models) and an *X* atom (Cl1 in the present models) in the same mirror plane. The bond strength of this short bond in the unmodulated high-temperature structure has been taken as a measure for the propensity of the compound to form modulated structures at low temperatures (Fabry & Perez-Mato, 1994 ▸).

Analysis of the interatomic distances of model D_r_ shows that they are in agreement with previous studies on similar compounds (Friese *et al.*, 2000 ▸), and that they support the interpretation given by Fabry & Perez-Mato (1994 ▸); see 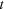-plots of distances and bond angles in the supplementary material.

The present model gives displacement modulations of Rb1 and Cl1 that are in-phase with each other (Fig. 5 ▸). Consequently, the very short Rb1–Cl1 distance hardly varies with phase 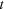 of the modulation. Instead, the strain of this contact is resolved by the modulated third-order anharmonic ADPs.

## 4. Conclusions   

A combination of structure refinements, analysis of the superspace MEM density and interpretation of difference-Fourier maps has been used to characterize the incommensurate modulation of Rb
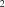
ZnCl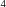 at a temperature of *T* = 196 K, close to the lock-in transition at 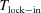 = 192 K. The basic characteristics of the modulation are a displacement modulation that contains contributions of Fourier coefficients up to fifth order.

A modulation of the ADPs is found to be an intrinsic part of the modulation. That is, the harmonic ADPs are modulated with up to second-order Fourier coefficients and the third-order anharmonic ADPs are modulated with Fourier coefficients up to fifth order, while the basic structure or average components of the third-order anharmonic ADPs are zero.

Model D_r_, which includes modulated ADPs and modulated third-order anharmonic ADPs, provides different values for the parameters of the displacement modulation than model A, which lacks any modulation of ADPs. Modulations of ADPs are thus essential for the correct description of the displacement modulation functions.

The MEM density gives an excellent description of the displacement modulations of the atoms by means of the 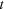-dependencies of the traces of the centers-of-charge of the atoms. These traces coincide with the displacement modulation functions of the atoms in model D_r_ but not in model A, providing further support for the necessity of modulated ADPs in the structure model.

Modulations of the ADPs and anharmonic ADPs are visible in the MEM density as variations of the distributions of the density about their average value, as exemplified by the traces of the local maxima of the MEM density around the positions of the atoms. A quantitative interpretation of the MEM density is made difficult by the finite resolution of this map, which limits the accuracy of positions to ∼ 0.01 Å.

Structure refinements may lead to a quantitative description of the modulation, but the introduction of the required model parameters readily leads to correlated parameters. Nevertheless, with the extensive data set available in the present study, we have been able to obtain significant values for higher-order Fourier coefficients of the displacive modulation functions and for modulated parameters of the harmonic ADPs and the third-order anharmonic ADPs.

The results suggest that modulated harmonic ADPs and modulated third-order anharmonic ADPs form an intrinsic part, however small, of incommensurately modulated structures.

For Rb
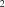
ZnCl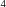 we could show that the modulation fails to provide clear evidence for a soliton wave as the principal shape of the modulation functions (Aramburu *et al.*, 2006 ▸). Instead, an extended theory will be necessary which includes the effects of modulated (an)harmonic ADPs to understand the modulations in *A* 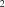 *BX* 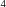 compounds.

## Supplementary Material

Crystal structure: contains datablock(s) global, I, II. DOI: 10.1107/S0108768111013814/bp5035sup1.cif

Structure factors: contains datablock(s) I. DOI: 10.1107/S0108768111013814/bp5035Isup2.hkl

Structure factors: contains datablock(s) II. DOI: 10.1107/S0108768111013814/bp5035IIsup3.hkl

Extra tables and figures. DOI: 10.1107/S0108768111013814/bp5035sup4.pdf

## Figures and Tables

**Figure 8:**
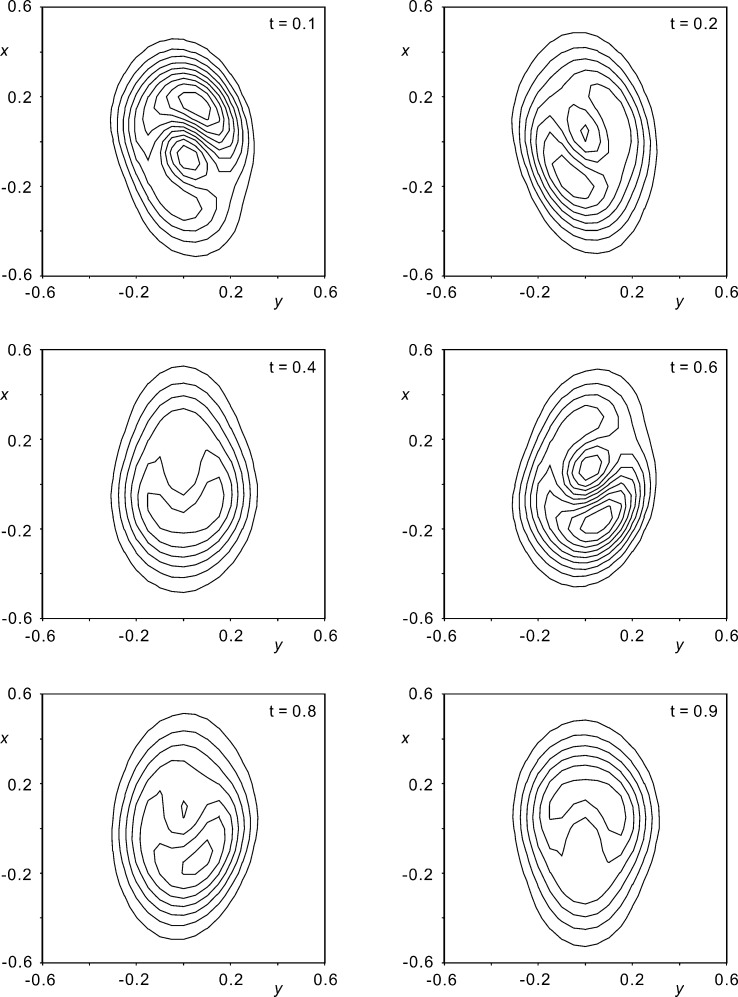
The joint probability distribution function at the site of Rb2 for selected 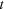 values for model D_r_. The contour interval is 1 e Å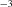. Over the selected map region the minimum density is −0.66 e Å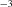 and the maximum density is 9.8 e Å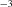.

**Table 1:** Experimental and crystallographic data

Crystal data	
Chemical formula	Rb_2_ZnCl_4_
*M*_r_	378.1
Crystal system, superspace group	Orthorhombic, *Pmcn*(00_3_)*ss* 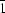
*T* (K)	196
Wavevectors	**q** = 0.31600c*
*a*, *b*, *c* ()	7.2405 (6), 12.630 (3), 9.2067 (6)†
*V* (^3^)	841.9 (3)
(mm^1^)	6.20
*Z*	4
Radiation type	Synchrotron (Hasylab, Hamburg, Germany)
Wavelength ()	0.5000
Crystal dimensions (mm)	0.10 0.10 0.12
	
Data collection	
Diffractometer	Huber four-circle (Kappa geometry)
Detector	marCCD165
[sin()/]_max_ (^1^)	0.86
()	0.3
()	0.3
Exposure times (s)	2, 8, 64
Absorption correction	*SADABS*
*T*_min_, *T*_max_	0.5143, 0.7506
Linear absorption coefficient (mm^1^)	6.08
Criterion of observability	*I* > 3(*I*)
*R*_int_(obs/all)	0.029/0.032
No. of measured reflections	75610
No. of unique reflections (obs/all)	6245/19956
No. of main reflections (obs/all)	1825/1956
No. of first-order satellites (obs/all)	2617/3517
No. of second-order satellites (obs/all)	1088/3803
No. of third-order satellites (obs/all)	392/3261
No. of fourth-order satellites (obs/all)	86/3870
No. of fifth-order satellites (obs/all)	237/3549
Average redundancy	3.789
	
Refinement (model D_r_)	
*R_F_*, *wR*(*F* ^2^), *S*	0.0563, 0.0705, 3.28
	
MEM	
Number of pixels	72 128 96 48
Pixel size (^3^)	0.100 0.099 0.096
*R_F_*, *wR*(*F* ^2^)	0.0112, 0.0225

† All calculations have been performed with the lattice parameters by Aramburu *et al.* (2006) ▸; see 2.1.

**Table 2:** Number of parameters for the different models *C_ijk_* and *D_ijkl_* represent the third- and fourth-order anharmonic ADPs. The models are defined in the text.

	Model A	Model B	Model C_r_	Model C	Model D_r_
*x* ^0^, *y* ^0^, *z* ^0^	13	13	13	13	13
*U* ^*ij*^	26	26	26	26	26
*C_ijk_*			0	0	0
*D_ijkl_*					33
Modulation of *x*, *y*, *z*	100	100	100	100	100
Modulation of *U* ^*ij*^		84	84	84	84
Modulation of *C_ijk_*			132	244	132
Scale	1	1	1	1	1
Total	140	224	356	468	389

**Table 3:** Amplitudes of the displacement modulation functions of model *A* (relative coordinates multiplied by 10^5^)

Atom	*n*	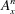	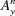	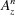	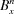	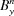	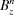
Rb1	1	1224 (1)	0	0	1215 (1)	0	0
	2	0	43 (9)	82 (10)	0	242 (8)	39 (10)
	3	291 (14)	0	0	35 (15)	0	0
	4	0	380 (30)	140 (40)	0	200 (30)	90 (40)
	5	68 (18)	0	0	38 (18)	0	0
Rb2	1	1713 (9)	0	0	140 (7)	0	0
	2	0	27 (6)	55 (8)	0	33 (6)	15 (8)
	3	103 (13)	0	0	97 (13)	0	0
	4	0	70 (20)	160 (40)	0	40 (20)	230 (40)
	5	161 (14)	0	0	70 (19)	0	0
Zn	1	998 (8)	0	0	329 (7)	0	0
	2	0	30 (7)	9 (9)	0	22 (7)	85 (9)
	3	25 (13)	0	0	99 (13)	0	0
	4	0	50 (30)	20 (40)	0	20 (30)	110 (50)
	5	108 (16)	0	0	11 (17)	0	0
Cl1	1	4250 (40)	0	0	660 (30)	0	0
	2	0	43 (18)	80 (20)	0	209 (19)	10 (20)
	3	400 (50)	0	0	240 (50)	0	0
	4	0	300 (60)	260 (80)	0	210 (60)	110 (70)
	5	110 (70)	0	0	140 (70)	0	0
Cl2	1	760 (30)	0	0	5480 (40)	0	0
	2	0	27 (17)	90 (20)	0	98 (16)	60 (30)
	3	380 (50)	0	0	1240 (50)	0	0
	4	0	10 (50)	150 (60)	0	110 (50)	150 (70)
	5	100 (70)	0	0	330 (80)	0	0
Cl3	1	554 (16)	2 (13)	1094 (15)	1939 (17)	2635 (15)	1334 (15)
	2	65 (18)	26 (15)	125 (17)	126 (18)	57 (16)	35 (17)
	3	230 (20)	80 (20)	30 (30)	330 (20)	535 (19)	320 (30)
	4	10 (40)	20 (40)	140 (50)	300 (40)	60 (30)	250 (50)
	5	140 (30)	0 (30)	90 (40)	100 (30)	250 (30)	10 (40)

**Table 4:** Quality of the fit to the diffraction data after refinements of models of increasing complexity Given are *R_F_* values of each order (|*m*|) of reflections, the number of parameters, _max_, _min_, the number of observed reflections *N*(obs) and the number of reflections N* with *I* > 5(*I*). Column 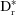 gives *R* values of model D_r_ calculated for the N* reflections. Model A includes displacement modulations, model B adds modulations of harmonic ADPs, model C incorporates modulations of third-order ADPs while model C_r_ is restricted to significant third-order ADP parameters, and model D_r_ adds basic structure parameters for fourth-order anharmonic ADPs. For details see 2.2.

Present data	Model A	Model B	Model C_r_	Model C	Model D_r_	*N*(obs)	Model 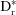	N*
All	0.1047	0.0698	0.0634	0.0633	0.0563	6245	0.0525	5145
*m* = 0	0.0776	0.0606	0.0561	0.0560	0.0493	1825	0.0487	1773
|*m*| = 1	0.1227	0.0703	0.0651	0.0650	0.0561	2617	0.0537	2358
|*m*| = 2	0.2388	0.1218	0.0988	0.0963	0.0969	1088	0.0787	683
|*m*| = 3	0.4807	0.2565	0.2045	0.2030	0.2003	392	0.1708	215
|*m*| = 4	0.6203	0.3149	0.2890	0.3046	0.2987	86	0.2023	12
|*m*| = 5	0.3434	0.2137	0.1757	0.1771	0.1619	237	0.1278	104
No. of parameters	140	224	356	468	389			
_max_ (e^3^)	4.85	2.11	2.17	2.18	1.71		1.74	
_min_ (e^3^)	3.74	2.61	2.09	2.09	1.78		1.71	

**Table 5:** *R_F_* values on the Aramburu data of models of increasing complexity, after refinement of the scale parameter, the extinction coefficient, the ADP parameters and the atomic coordinates Modulation parameters were kept fixed at their values obtained by refinements against the present data.

Published data	Model A	Model B	Model C_r_	Model C	Model D_r_	*N*(obs)
All	0.0834	0.0917	0.0912	0.0912	0.0912	1695
*m* = 0	0.0784	0.0837	0.0828	0.0827	0.0826	778
|*m*| = 1	0.0733	0.0855	0.0892	0.0900	0.0896	473
|*m*| = 2	0.2281	0.3820	0.3564	0.3605	0.3569	251
|*m*| = 3	0.4636	0.3049	0.2989	0.2859	0.2976	53
|*m*| = 4						
|*m*| = 5	0.3647	0.2623	0.2807	0.2667	0.2867	140

**Table 6:** Amplitudes of the displacement modulation functions of model D_r_ (relative coordinates multiplied by 10^5^)

Atom	*n*	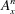	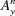	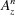	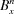	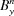	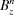
Rb1	1	1111 (8)	0	0	1100 (9)	0	0
	2	0	21 (9)	94 (6)	0	171 (8)	10 (10)
	3	175 (16)	0	0	191 (16)	0	0
	4	0	81 (16)	0 (20)	0	103 (15)	120 (20)
	5	101 (9)	0	0	94 (9)	0	0
Rb2	1	1574 (8)	0	0	149 (6)	0	0
	2	0	23 (3)	58 (4)	0	10 (3)	16 (5)
	3	148 (15)	0	0	49 (8)	0	0
	4	0	8 (12)	40 (18)	0	27 (11)	59 (18)
	5	85 (15)	0	0	29 (10)	0	0
Zn	1	958 (7)	0	0	308 (6)	0	0
	2	0	29 (3)	11 (5)	0	26 (6)	82 (5)
	3	21 (16)	0	0	130 (8)	0	0
	4	0	42 (13)	30 (20)	0	21 (16)	40 (20)
	5	89 (8)	0	0	12 (8)	0	0
Cl1	1	3770 (30)	0	0	580 (30)	0	0
	2	0	15 (10)	60 (15)	0	143 (19)	10 (20)
	3	80 (50)	0	0	160 (50)	0	0
	4	0	80 (40)	70 (40)	0	20 (30)	20 (40)
	5	170 (70)	0	0	60 (70)	0	0
Cl2	1	770 (30)	0	0	5100 (40)	0	0
	2	0	4 (11)	77 (12)	0	68 (9)	40 (19)
	3	110 (40)	0	0	560 (50)	0	0
	4	0	0 (30)	20 (30)	0	70 (30)	10 (40)
	5	90 (80)	0	0	70 (80)	0	0
Cl3	1	501 (15)	67 (12)	1022 (10)	1811 (15)	2427 (14)	1227 (12)
	2	5 (18)	31 (14)	100 (15)	81 (17)	28 (13)	12 (17)
	3	40 (30)	40 (30)	180 (30)	184 (14)	335 (18)	10 (30)
	4	20 (20)	60 (20)	50 (30)	120 (20)	79 (19)	100 (30)
	5	100 (30)	40 (40)	100 (40)	80 (30)	190 (30)	10 (30)

## Footnotes

1 Supplementary data for this paper are available from the IUCr electronic archives (Reference: BP5035). Services for accessing these data are described at the back of the journal.
