# Supplementary material for: Modulated anharmonic ADPs are intrinsic to aperiodic crystals: a case study on incommensurate Rb2ZnCl4
Source: Acta Crystallogr B. 2011 May 14;67(Pt 3):205–17. doi: 10.1107/S0108768111013814 (PMC3098556; doi:10.1107/S0108768111013814)
Supplement: Supplementary file 4 [file b-67-00205-sup4.pdf]

Incommensurately modulated structure of Rb_2ZnCl_4 studied by the Maximum Entropy Method (MEM)

Supplementary Material

Liang Li, Alexander Wölfel, Andreas Schönleber, Swastik Mondal, Antoine M. M. Schreurs, Loes M. J. Kroon-Batenburg, Sander van Smaalen

December 28, 2010

Table 1: Basic positions (relative coordinates) of the crystallographically independent atoms in model A and model D_r.

	Model A			Model D_r		
Atom	x^0	y^0	z^0	x^0	y^0	z^0
Rb1	0.25	0.40659(8)	0.62976(8)	0.25	0.40660(4)	0.62969(4)
Rb2	0.25	0.81909(4)	0.48680(6)	0.25	0.81921(2)	0.48673(3)
Zn	0.25	0.42189(5)	0.22339(7)	0.25	0.42179(3)	0.22344(3)
Cl1	0.25	0.42044(14)	-0.01861(17)	0.25	0.41962(8)	-0.01837(9)
Cl2	0.25	0.58417(12)	0.32210(17)	0.25	0.58430(6)	0.32169(9)
Cl3	0.00210(13)	0.33922(10)	0.31206(13)	0.00171(7)	0.33945(6)	0.31245(8)

Table 2: Basic-structure parameters (Å^2) and modulation amplitudes (Å^2) of the harmonic ADPs for model D$_r$. Values have been multiplied by 10^5; standard uncertainties are given in parentheses.

Atom	Function	U_{11}	U_{22}	U_{33}	U_{12}	U_{13}	U_{23}
Rb1	Basic	4160(40)	6340(70)	1760(50)	0	0	-170(30)
	sin1	0	0	0	444(16)	-101(12)	0
	cos1	0	0	0	648(15)	-389(12)	0
	sin2	120(20)	470(40)	50(30)	0	0	-112(18)
	cos2	-110(20)	20(30)	10(20)	0	0	30(20)
Rb2	Basic	3410(40)	2020(40)	1540(40)	0	0	-80(19)
	sin1	0	0	0	-382(10)	294(10)	0
	cos1	0	0	0	113(9)	-45(9)	0
	sin2	3(17)	0(20)	-16(19)	0	0	-20(13)
	cos2	312(16)	50(20)	50(19)	0	0	-9(13)
Zn	Basic	2085(15)	1969(19)	1426(18)	0	0	-21(8)
	sin1	0	0	0	76(10)	-79(9)	0
	cos1	0	0	0	-16(10)	-15(9)	0
	sin2	-1(16)	-20(20)	-10(20)	0	0	5(17)
	cos2	48(16)	40(20)	-10(20)	0	0	-30(15)
Cl1	Basic	7660(60)	3730(50)	1390(40)	0	0	-430(20)
	sin1	0	0	0	1750(50)	160(40)	0
	cos1	0	0	0	-180(50)	-280(40)	0
	sin2	-470(80)	-70(70)	0(60)	0	0	-50(40)
	cos2	1520(90)	-70(70)	20(60)	0	0	-80(40)
Cl2	Basic	7500(70)	2320(40)	2170(40)	0	0	-680(20)
	sin1	0	0	0	100(50)	260(50)	0
	cos1	0	0	0	-760(40)	190(40)	0
	sin2	-50(80)	0(60)	20(60)	0	0	10(40)
	cos2	-2180(100)	60(60)	50(70)	0	0	-80(40)
Cl3	Basic	2730(50)	6800(100)	3250(80)	-2240(50)	-530(50)	1810(60)
	sin1	-80(30)	-490(60)	850(40)	310(30)	-60(20)	270(40)
	cos1	-490(20)	-450(50)	-720(40)	580(30)	240(20)	-480(30)
	sin2	130(30)	350(80)	290(60)	-310(40)	-190(30)	330(50)
	cos2	-330(30)	-1620(70)	-120(50)	770(40)	230(30)	-690(60)

Table 3: Values of fourth-order anharmonic ADPs of model D_r (D_{ijkl} multiplied by 10^9).

D_{ijkl}	Rb1	Rb2	Cl3
D_{1111}	-150(30)	-90(20)	-70(30)
D_{1112}	0	0	-16(12)
D_{1113}	0	0	-32(13)
D_{1122}	-53(5)	-34(3)	-28(8)
D_{1123}	-2(3)	-0.3(18)	-12(6)
D_{1133}	-47(6)	-64(4)	-73(9)
D_{1222}	0	0	6(8)
D_{1223}	0	0	4(5)
D_{1233}	0	0	-1(5)
D_{1333}	0	0	-13(12)
D_{2222}	-62(6)	-27(3)	-25(12)
D_{2223}	2(3)	-0.7(11)	4(7)
D_{2233}	21(3)	12.1(14)	44(6)
D_{2333}	8(4)	3(2)	20(9)
D_{3333}	-98(14)	-121(10)	-210(20)

Table 4: Modulation amplitude (multiplied by 10^8) of third-order anharmonic ADPs (C_{ijk}) of model D_r (part 1).

Atom	Function	C_{111}	C_{112}	C_{113}	C_{122}	C_{123}	C_{133}	C_{222}	C_{223}	C_{233}	C_{333}
Rb1	sin1	180(30)	0	0	93(8)	0	0	0	0	0	0
	cos1	320(30)	0	0	38(8)	-12(5)	-13(9)	0	0	0	0
	sin2	0	56(18)	0	0	0	0	31(16)	-12(9)	18(11)	0
	cos2	0	113(16)	-19(18)	0	0	0	78(15)	-13(10)	36(9)	-30(30)
	sin3	360(70)	0	0	70(20)	0	0	0	0	0	0
	cos3	-250(70)	0	0	40(20)	0	0	0	0	0	0
Rb2	sin1	-350(20)	0	0	-15(4)	0	0	0	0	0	0
	cos1	-40(20)	0	0	0	0	0	0	0	0	0
	sin2	0	0	0	0	0	0	0	0	0	0
	cos2	0	-47(11)	27(14)	0	0	0	0	0	0	0
	sin3	50(60)	0	0	7(14)	0	20(20)	0	0	0	0
	cos3	0	0	0	43(14)	0	0	0	0	0	0
	sin5	-300(80)	0	0	0	0	-30(30)	0	0	0	0
	cos5	0	0	0	0	0	0	0	0	0	0

Table 5: Modulation amplitude (multiplied by 10^8) of third-order anharmonic ADPs (C_{ijk}) of model D_r (part 2).

Atom	Function	C_{111}	C_{112}	C_{113}	C_{122}	C_{123}	C_{133}	C_{222}	C_{223}	C_{233}	C_{333}
Zn	sin1	-90(20)	0	0	0	0	0	0	0	0	0
	cos1	-50(20)	0	0	0	0	0	0	0	0	0
	sin2	0	0	0	0	0	0	0	0	0	0
	cos2	0	0	0	0	0	0	-14(12)	0	0	0
	sin3	180(70)	0	0	-9(17)	0	0	0	0	0	0
	cos3	0	0	0	23(15)	0	0	0	0	0	0
Cl1	sin1	-2970(170)	0	0	-10(20)	24(16)	-80(30)	0	0	0	0
	cos1	-330(190)	0	0	-30(20)	-22(17)	0	0	0	0	0
	sin2	0	0	100(60)	0	0	0	0	0	0	0
	cos2	0	260(50)	0	0	0	0	30(30)	-27(17)	20(20)	60(60)
	sin3	-150(30)	0	0	0	0	0	0	0	0	0
	cos3	80(30)	0	0	110(50)	0	0	0	0	0	0
	sin5	-1000(400)	0	0	0	0	0	0	0	0	0
	cos5	1000(5000)	0	0	0	0	0	0	0	0	0

Table 6: Modulation amplitude (multiplied by 10^8) of third-order anharmonic ADP (C_{ijk}) of model D_r (part 3).

Atom	Function	C_{111}	C_{112}	C_{113}	C_{122}	C_{123}	C_{133}	C_{222}	C_{223}	C_{233}	C_{333}
Cl2	sin1	300(200)	0	0	0	0	0	0	0	0	0
	cos1	-1520(160)	0	0	-35(19)	0	-70(40)	0	0	0	0
	sin2	0	-120(50)	60(30)	0	0	0	0	0	0	0
	cos2	0	0	-90(60)	0	0	0	0	-19(16)	0	0
	sin3	400(200)	0	0	0	0	0	0	0	0	0
	cos3	2200(300)	0	0	70(40)	0	90(80)	0	0	0	0
	sin5	-1100(500)	0	0	-160(90)	70(100)	-210(170)	0	0	0	0
	cos5	-2900(600)	0	0	0	0	30(180)	0	0	0	0
Cl3	sin1	-80(50)	100(30)	0	-90(20)	0	0	140(30)	81(150)	0	0
	cos1	250(40)	-210(20)	120(20)	234(18)	115(13)	0	-360(20)	-162(15)	109(15)	0
	sin2	-70(60)	30(20)	40(30)	-46(19)	0	-40(30)	40(20)	0	-23(18)	-50(40)
	cos2	-150(60)	140(30)	90(30)	-130(20)	-72(18)	50(30)	170(30)	75(17)	0	-110(40)
	sin3	180(110)	-100(50)	-150(50)	130(30)	0	0	-130(50)	-100(30)	40(40)	-290(80)
	cos3	0	0	60(50)	-190(30)	-130(30)	0	260(40)	60(30)	30(30)	260(8)
	sin5	230(160)	-160(90)	-10(90)	110(60)	0	0	-280(90)	-70(70)	-20(80)	20(180)
	cos5	60(180)	-150(90)	0	120(60)	0	-80(80)	0	90(70)	100(70)	-90(170)

Table 7: Various models refined against present data. Given are the R values for each order of reflections, the number of parameters, $(\Delta\rho)_{max}$, $(\Delta\rho)_{min}$ and the values of the joint probability distribution function (j.P.D.F.) for each atom. The models differ in the atoms for which fourth-order anharmonic ADPs have been refined: Model D1 (Zn), D2 (Rb1), D3 (Rb2), D4 (Zn, Rb1), D5 (Rb1, Rb2), D6 (Zn, Cl1, Cl2), D_r (Rb1, Rb2, Cl3), D8 (Rb1, Rb2, Cl1, Cl3), D9 (Rb1 Rb2 Zn Cl3), D10 (Rb1, Rb2, Cl1, Cl2, Cl3) and D11 (Rb1, Rb2, Zn, Cl1, Cl2, and Cl3).

	D1	D2	D3	D4	D5	D6	D_r	D8	D9	D10	D11
All	0.0605	0.0620	0.0598	0.0582	0.0575	0.0597	0.0563	0.0554	0.0486	0.0549	0.0468
$m = 0$	0.0521	0.0545	0.0522	0.0495	0.0497	0.0516	0.0493	0.0484	0.0396	0.0482	0.0388
$\|m\| = 1$	0.0638	0.0639	0.0616	0.0620	0.0593	0.0623	0.0561	0.0550	0.0513	0.0537	0.0472
$\|m\| = 2$	0.0990	0.0982	0.0986	0.0983	0.0974	0.0982	0.0969	0.0969	0.0963	0.0969	0.954
$\|m\| = 3$	0.2040	0.2035	0.2036	0.2024	0.2031	0.2033	0.2003	0.2005	0.1996	0.2001	0.1967
$\|m\| = 4$	0.2901	0.2882	0.2903	0.2904	0.2908	0.2911	0.2987	0.2992	0.3133	0.2972	0.3025
$\|m\| = 5$	0.1744	0.1734	0.1723	0.1730	0.1707	0.1740	0.1619	0.1623	0.1664	0.1625	0.1664
No. of parameters	365	365	365	374	374	383	389	398	398	407	416
$(\Delta\rho)_{max}$ (e Å^{-3})	2.11	2.11	1.75	2.04	1.78	2.03	1.71	1.76	1.50	1.77	1.59
$(\Delta\rho)_{min}$ (e Å^{-3})	-2.06	-2.06	-1.86	-2.03	-1.78	-2.02	-1.78	-1.78	-1.39	-1.80	-1.47
$(j.P.D.F)_{min}$ (e Å^{-3})											
Rb1	0	-0.01	-0.00	-0.17	-0.21	0	-0.21	-0.40	-10.80	-0.19	-20.80
Rb2	0	-0.02	0	0	-1.05	-0.02	-1.04	-2.91	-67.54	-5.67	-145.24
Zn	-1.50	0.00	-0.03	-17.72	0	-3.59	0	-0.01	-447.10	-0.15	-1042.14
Cl1	-0.08	-0.01	-0.07	-0.01	-0.02	-6.40	-0.05	-61.13	-0.08	-106.02	-378.06
Cl2	-0.04	-0.41	-0.05	0	0	-0.80	0	0	0	-1.09	-69.42
Cl3	-0.54	-0.57	-0.30	-0.53	-0.54	-0.55	-2.05	-3.44	-39.12	-4.38	-80.03

Table 8: Amplitudes (relative coordinates) of the displacement modulation function (multiplied by 10^5) after refinement of the model of Aramburu against Aramburu data.

Atom	n	A_x^n	A_y^n	A_z^n	B_x^n	B_y^n	B_z^n
Rb1	1	1250(40)	0	0	-1180(40)	0	0
	2	0	-33(18)	110(20)	0	-227(18)	20(20)
	3	-80(40)	0	0	10(40)	0	0
	5	129(18)	0	0	-140(20)	0	0
Rb2	1	1710(30)	0	0	-140(30)	0	0
	2	0	12(10)	-65(19)	0	12(10)	0(20)
	3	-100(30)	0	0	-120(30)	0	0
	5	132(15)	0	0	30(20)	0	0
Zn	1	1030(30)	0	0	330(30)	0	0
	2	0	-38(11)	2(9)	0	-6(10)	33(17)
	3	20(20)	0	0	-170(30)	0	0
	5	-108(15)	0	0	15(15)	0	0
Cl1	1	3980(90)	0	0	700(80)	0	0
	2	0	-110(40)	-10(40)	0	-130(30)	0(50)
	3	20(90)	0	0	120(70)	0	0
	5	390(50)	0	0	-20(60)	0	0
Cl2	1	820(80)	0	0	5420(90)	0	0
	2	0	-30(50)	40(50)	0	-80(30)	130(50)
	3	-710(100)	0	0	-1260(90)	0	0
	5	250(60)	0	0	250(70)	0	0
Cl3	1	580(50)	-60(30)	-1120(40)	-1860(50)	2690(30)	1440(50)
	2	50(50)	-10(30)	30(40)	40(30)	-40(30)	70(30)
	3	-160(40)	150(30)	170(60)	280(40)	-690(30)	-350(70)
	5	-260(30)	160(30)	210(20)	0(30)	170(30)	30(30)

Table 9: Quality of the fit to the Aramburu data after refinements of models of increasing complexity. Given are R values of each order ($|m|$) of reflections, the number of parameters, $(\Delta\rho)_{max}$, $(\Delta\rho)_{min}$ and the number of observed reflections N(obs).

Pulished data	Model A′	Model B′	Model C_r′	Model C′	Model D_r′	N(obs)
All	0.0827	0.0739	0.0726	0.0719	0.0717	1695
$m = 0$	0.0794	0.0763	0.0765	0.0770	0.0755	778
$\|m\| = 1$	0.0751	0.0479	0.0458	0.0444	0.0453	473
$\|m\| = 2$	0.2044	0.1452	0.1205	0.0968	0.1199	251
$\|m\| = 3$	0.3470	0.2957	0.1716	0.1103	0.1759	53
$\|m\| = 4$	–	–	–	–	–	–
$\|m\| = 5$	0.2100	0.1909	0.1658	0.1339	0.1651	140
No. of parameters	115	199	331	443	364	
$(\Delta\rho)_{max}$ (e Å^{-3})	2.74	2.49	2.27	2.28	2.35	
$(\Delta\rho)_{min}$ (e Å^{-3})	-2.05	-1.91	-1.86	-1.76	-1.79	

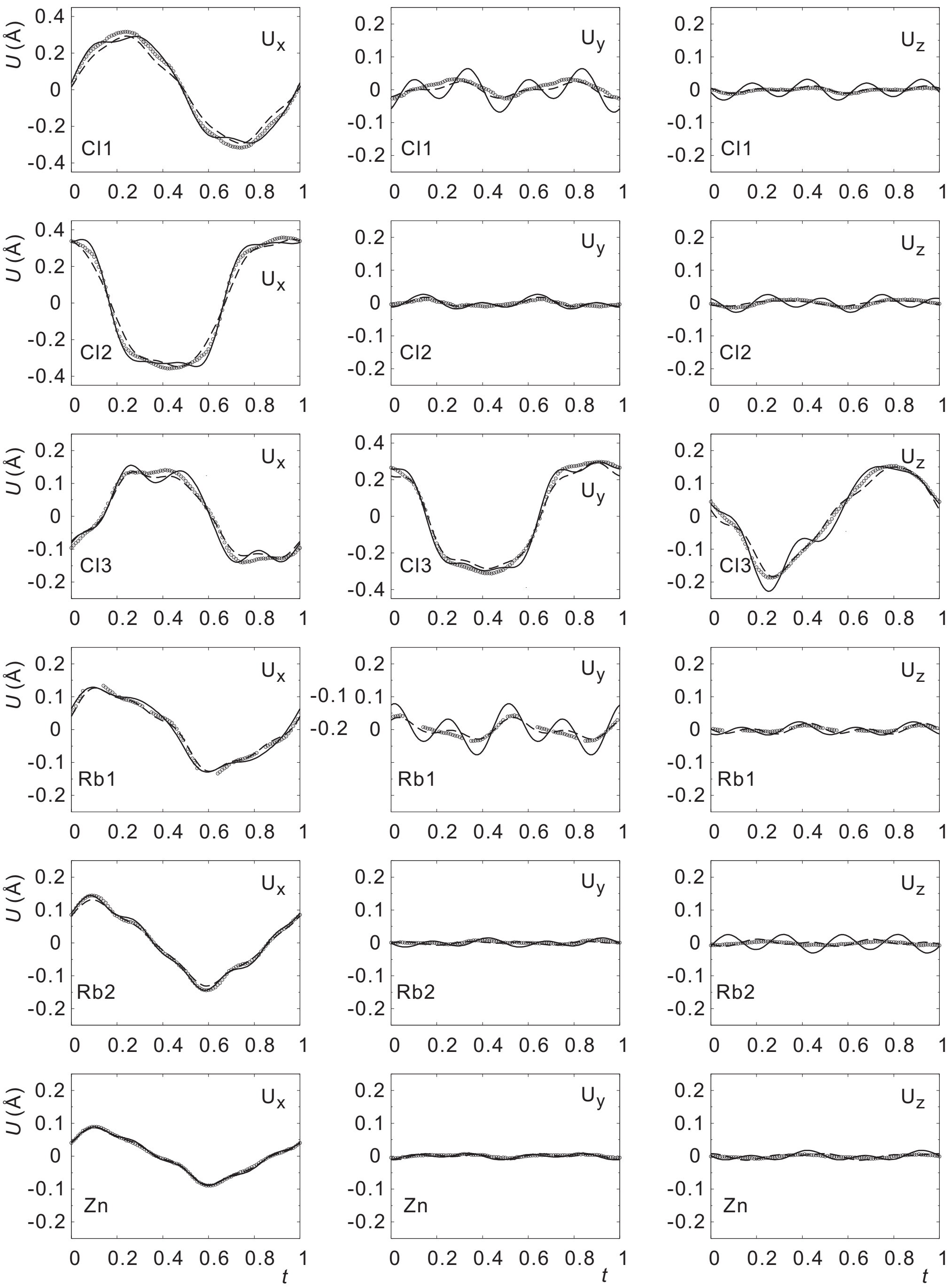

Figure 1: Modulation functions of the crystallographically independent atoms of Rb_2ZnCl_4 of model A (solid line) and model D_r (dashed line). Open circles reflect the center of charge in the MEM density. Displacements along x, y and z are given in Å.

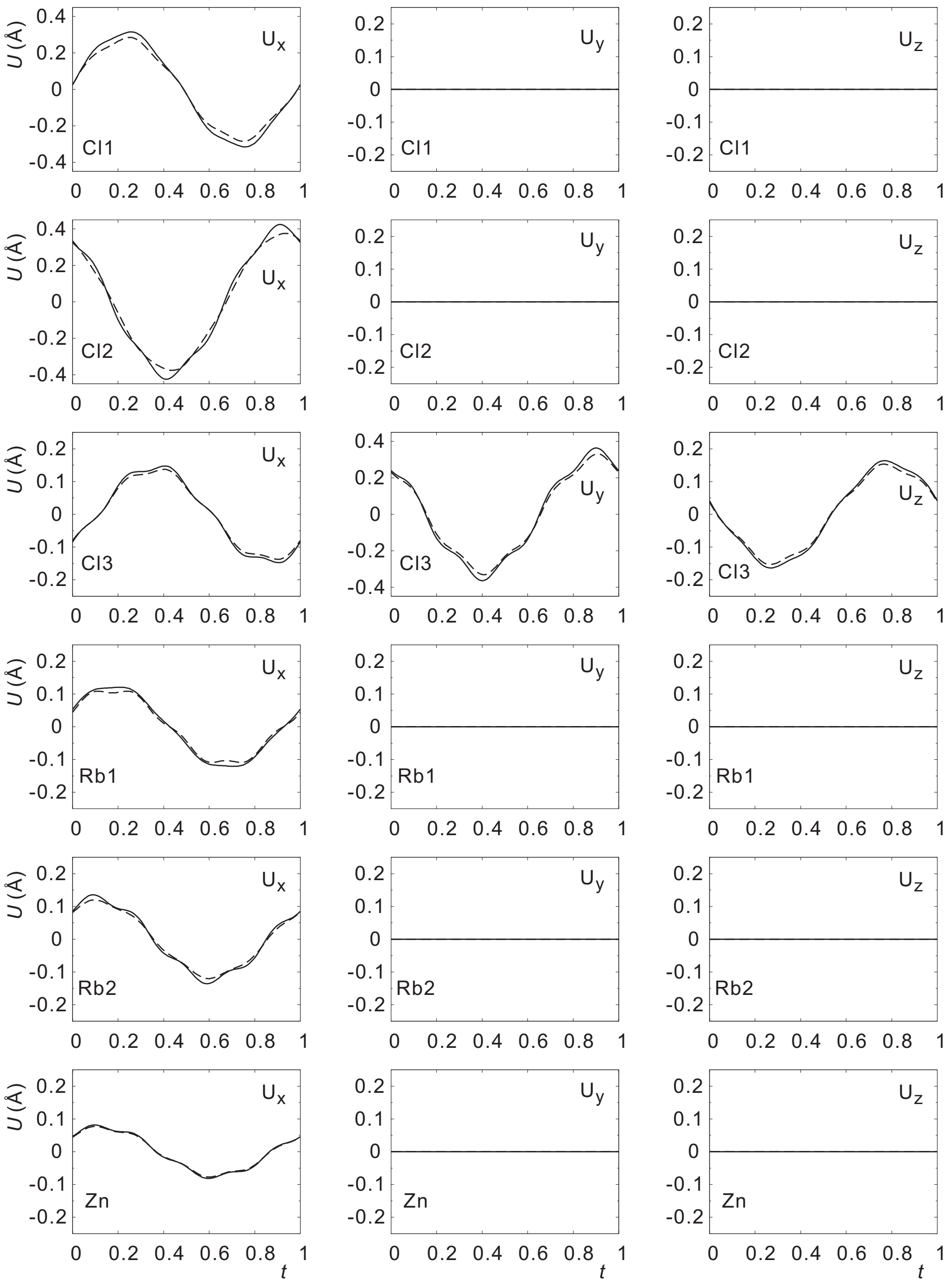

Figure 2: Modulation functions only corresponding to the first and fifth-order harmonics of the crystallographically independent atoms of Rb_2ZnCl_4 of model A (solid line) and model D_r (dashed line). Displacements along x, y and z are given in Å.

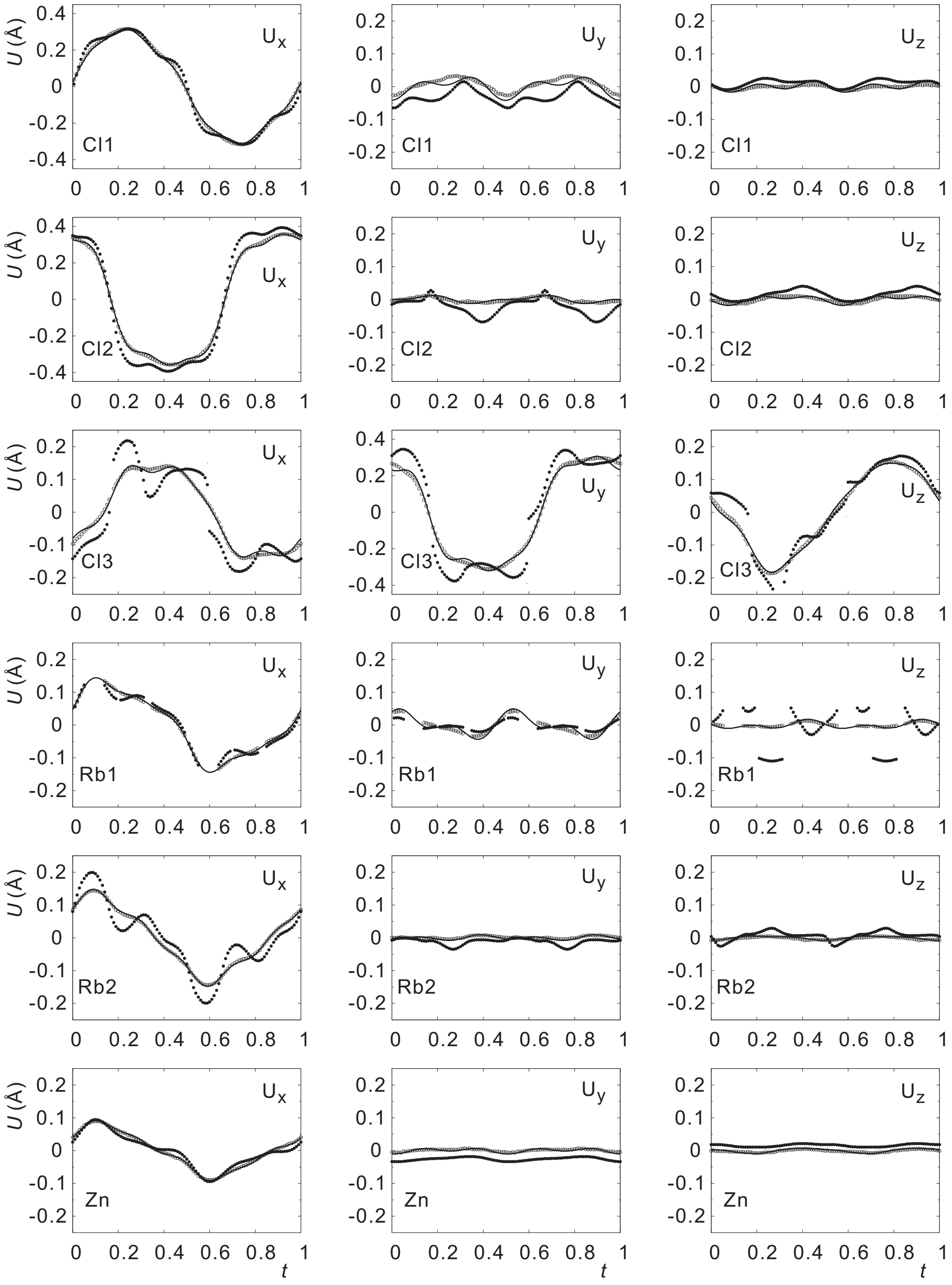

Figure 3: Modulation functions of the crystallographically independent atoms of Rb_2ZnCl_4. Solid lines are the modulation function from model B. Open circles reflect the center of charge, filled circles the local maxima of the MEM electron density. Displacement along x, y and z are given in Å.

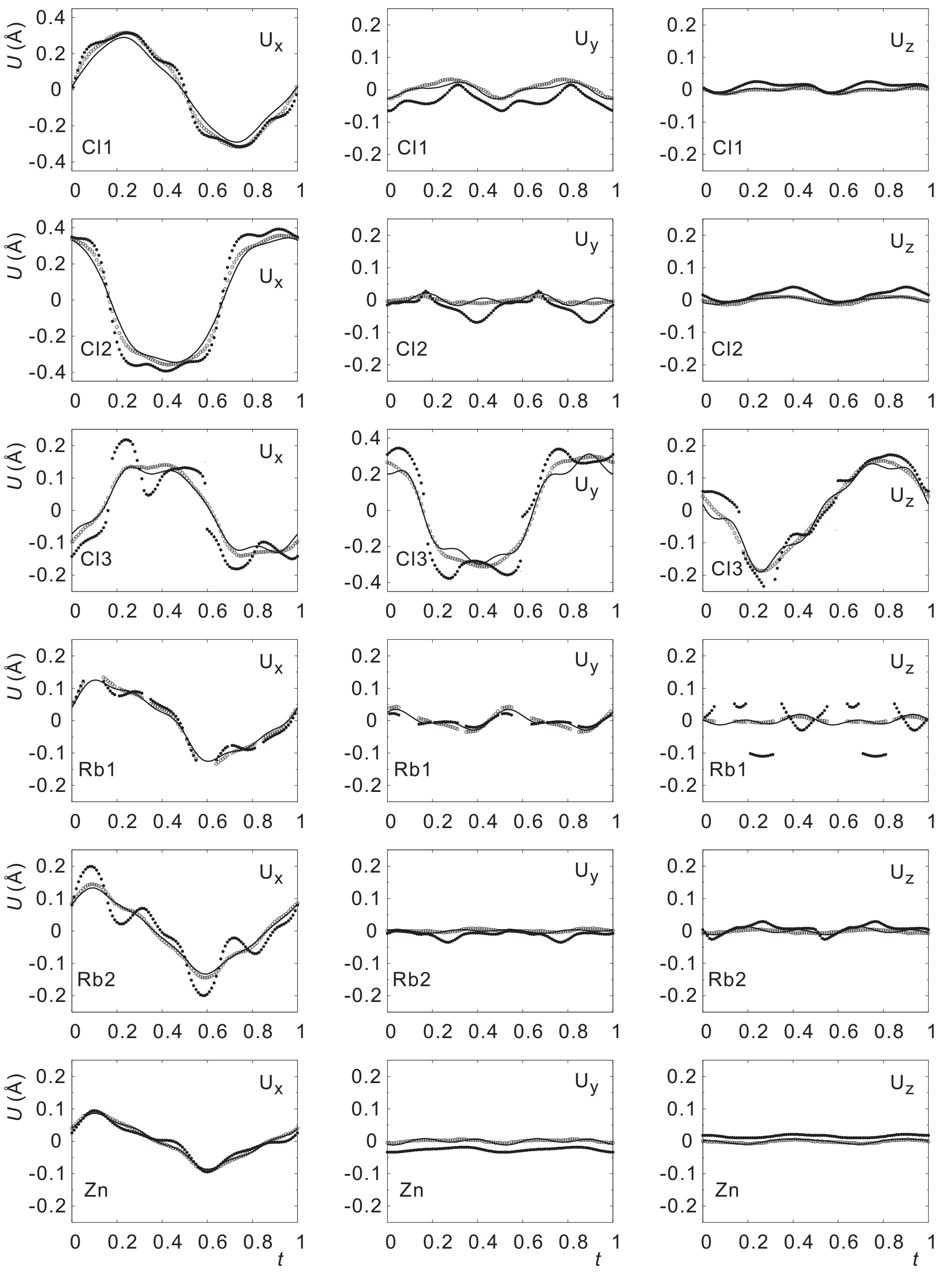

Figure 4: Modulation functions of the crystallographically independent atoms of Rb_2ZnCl_4. Solid lines are the modulation function from model C_r. Open circles reflect the center of charge, filled circles the local maxima of the MEM electron density. Displacements along x, y and z are given in Å.

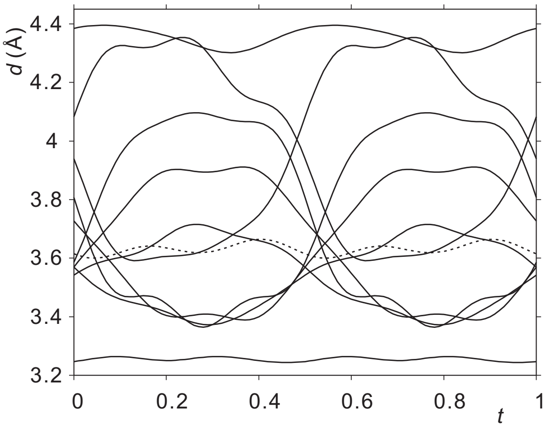

Figure 5: t-Plot of the distances between atom Rb1 and the eleven nearest neighbor Cl atoms for model D_r. The dashed line highlights one particular pair of atoms Rb1–Cl

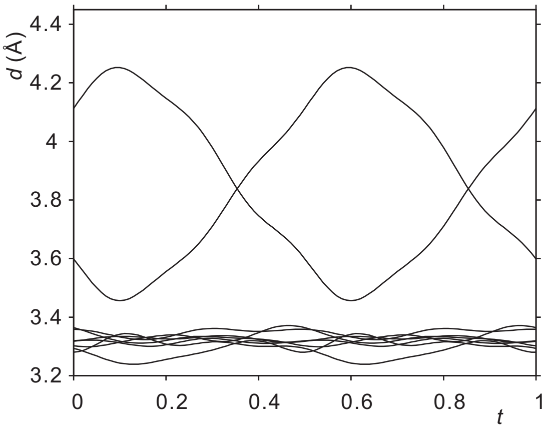

Figure 6: t-Plot of the distances between atom Rb2 and the nine nearest neighbor Cl atoms for model D_r.

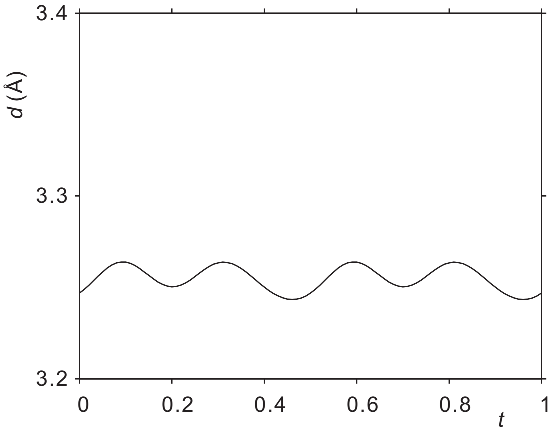

Figure 7: t-Plot of the distance Rb1–Cl1(i) for model D_r [symmetry code $(x_1, x_2, x_3 + 1, x_4)$].

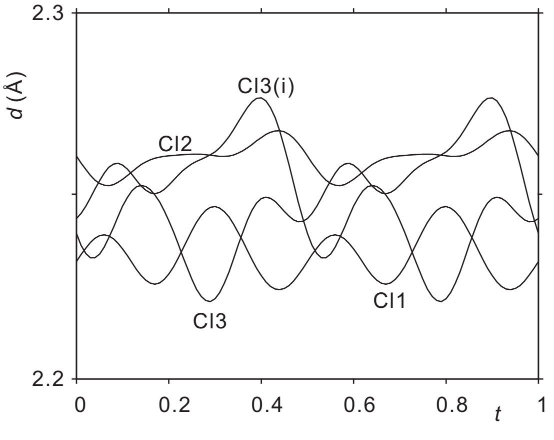

Figure 8: t-Plot of the distances between atom Zn and the surrounding atoms Cl1, Cl2, Cl3 and Cl3(i) for model D_r [symmetry code $(-x_1 + \frac{1}{2}, x_2, x_3, x_4 + \frac{1}{2})$].

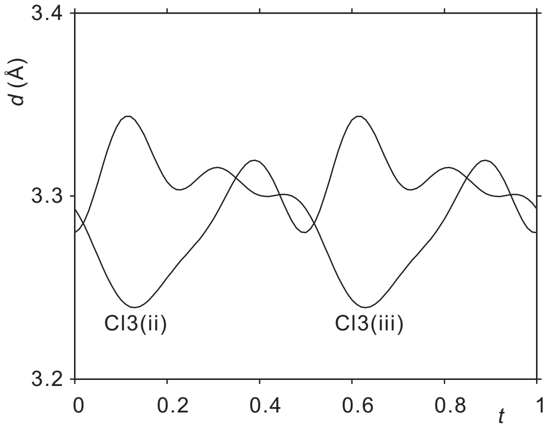

Figure 9: t-Plot of the distances between Rb2 and the two nearest Cl atoms for model D_r, Cl3(ii) [symmetry code $(-x_1, 1-x_2, 1-x_3, -x_4)$] and Cl3(iii) [symmetry code $(x_1 + \frac{1}{2}, 1-x_2, 1-x_3, -x_4+\frac{1}{2})$].

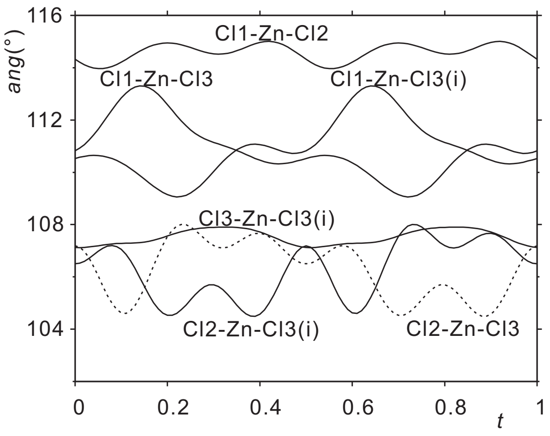

Figure 10: t-Plot of the bond angles Cl–Zn–Cl for model D_r. The dashed line highlights one particular triplet of atoms.

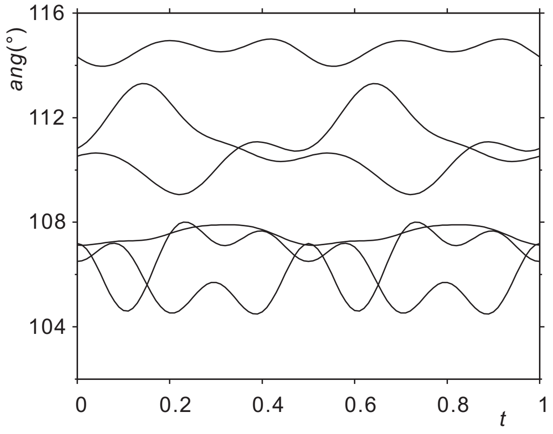

Figure 11: t-Plot of the bond angles Cl–Zn–Cl for model D_r.

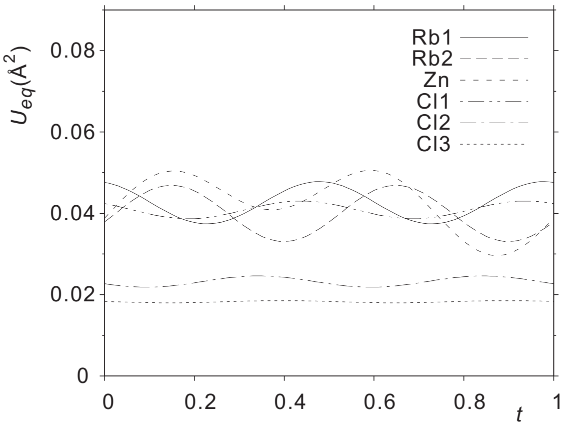

Figure 12: t-Plot of U_{eq} of all atoms for model D_r.

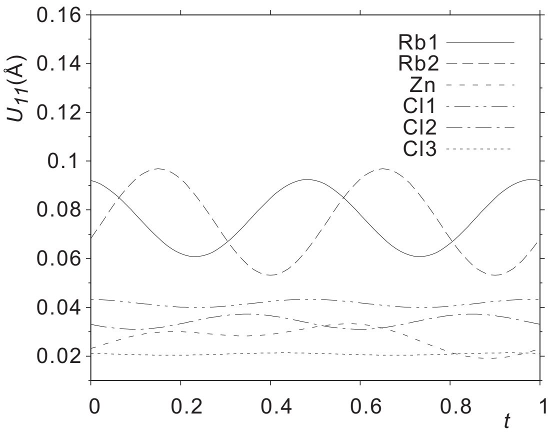

Figure 13: t-Plot of U_{11} of all atoms for model D_r.

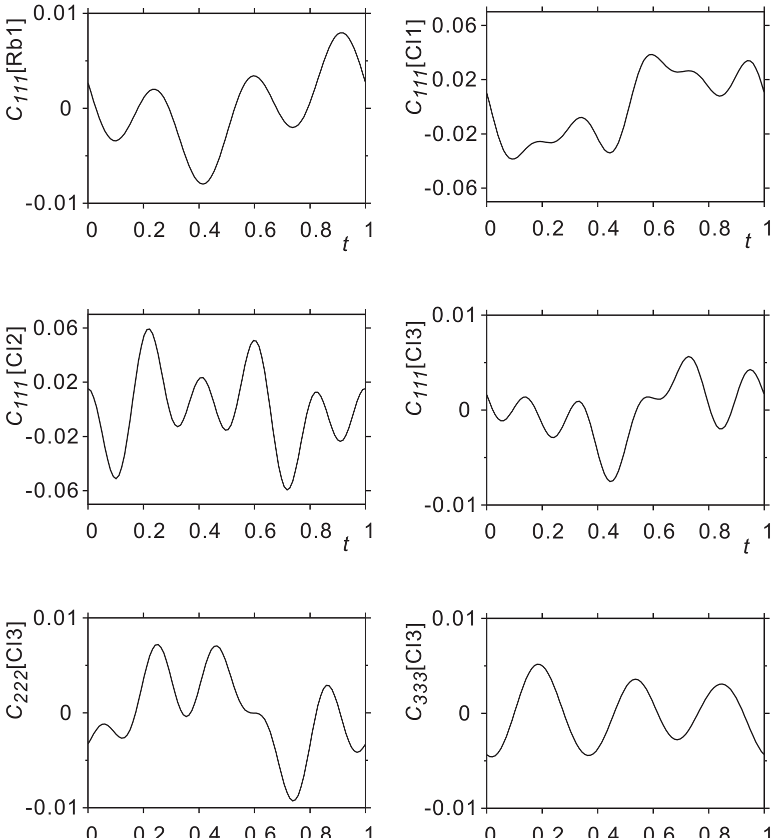

Figure 14: t-Plots of selected third-order anharmonic ADPs C_{ijk} of all atoms for model D_r.
